# Evaluation of multiple imputation approaches for handling missing covariate information in a case-cohort study with a binary outcome

**DOI:** 10.1186/s12874-021-01495-4

**Published:** 2022-04-03

**Authors:** Melissa Middleton, Cattram Nguyen, Margarita Moreno-Betancur, John B. Carlin, Katherine J. Lee

**Affiliations:** 1 Clinical Epidemiology & Biostatistics Unit, Murdoch Children’s Research Institute, Royal Children’s Hospital, 50 Flemington Rd, Parkville, Melbourne, VIC 3052 Australia; 2 Department of Paediatrics, The University of Melbourne, Parkville, Australia

**Keywords:** Multiple imputation, Case-cohort study, Simulation study, Missing data, Unequal sampling probability, Inverse probability weighting

## Abstract

**Background:**

In case-cohort studies a random subcohort is selected from the inception cohort and acts as the sample of controls for several outcome investigations. Analysis is conducted using only the cases and the subcohort, with inverse probability weighting (IPW) used to account for the unequal sampling probabilities resulting from the study design. Like all epidemiological studies, case-cohort studies are susceptible to missing data. Multiple imputation (MI) has become increasingly popular for addressing missing data in epidemiological studies. It is currently unclear how best to incorporate the weights from a case-cohort analysis in MI procedures used to address missing covariate data.

**Method:**

A simulation study was conducted with missingness in two covariates, motivated by a case study within the Barwon Infant Study. MI methods considered were: using the outcome, a proxy for weights in the simple case-cohort design considered, as a predictor in the imputation model, with and without exposure and covariate interactions; imputing separately within each weight category; and using a weighted imputation model. These methods were compared to a complete case analysis (CCA) within the context of a standard IPW analysis model estimating either the risk or odds ratio. The strength of associations, missing data mechanism, proportion of observations with incomplete covariate data, and subcohort selection probability varied across the simulation scenarios. Methods were also applied to the case study.

**Results:**

There was similar performance in terms of relative bias and precision with all MI methods across the scenarios considered, with expected improvements compared with the CCA. Slight underestimation of the standard error was seen throughout but the nominal level of coverage (95%) was generally achieved. All MI methods showed a similar increase in precision as the subcohort selection probability increased, irrespective of the scenario. A similar pattern of results was seen in the case study.

**Conclusions:**

How weights were incorporated into the imputation model had minimal effect on the performance of MI; this may be due to case-cohort studies only having two weight categories. In this context, inclusion of the outcome in the imputation model was sufficient to account for the unequal sampling probabilities in the analysis model.

**Supplementary Information:**

The online version contains supplementary material available at 10.1186/s12874-021-01495-4.

## Background

Epidemiological studies often collect large amounts of data on many individuals. Some of this information may be costly to analyse, for example biological samples. Furthermore, if there are a limited number of cases, data on all non-cases may provide little additional information to that provided by a subset [1]. In this context, investigators may opt to use a case-cohort study design, in which background covariate data and outcomes are collected on all participants and more costly exposures (e.g. metabolite levels) are collected on a smaller subset. An example of a cohort study adopting the case-cohort design is the Barwon Infant Study (BIS). This is a population-derived cohort study with a focus on non-communicable diseases and the biological processes driving them. Given that a number of investigations within BIS involve exposures collected through costly biomarker and metabolite analysis, for example serum vitamin D levels, the case-cohort design was implemented to minimise cost [2].

In the case-cohort design, a subset of the full cohort, hereafter termed the subcohort, is randomly selected from the inception cohort. This subcohort is used as the sample of controls for all subsequent investigations, with exposure data collected from the subcohort and all cases [3]. In such a study, the analysis is conducted on the subcohort and cases only, resulting in an unequal probability of selection into the analysis, with cases having probability of selection equal to1 and non-case subcohort members having a probability of selection less than 1. This unequal sampling should be accounted for in the analysis so as to avoid bias induced due to the oversampling of cases [4].

One way to view the case-cohort design, and to address the unequal sampling, is to treat it as a missing data problem, where the exposure data is ‘missing by design’. Standard practice in the analysis of case-cohort studies is to handle this missing exposure data using inverse probability weighting (IPW) based on the probability of selection into the analysis [1]. Additionally, case-cohort studies may be subject to unintended missing data in the covariates, and multiple imputation (MI) may be applied to address this missing data.

MI is a two-stage procedure in which missing values are first imputed by drawing plausible sets of values from the posterior distribution of the missing data given the observed data, to form multiple, say *m > 1,* complete datasets. In the second stage, the analysis is conducted on each of these *m* datasets as though they were fully observed, producing *m* estimates of the target estimands. An overall estimate for the parameter of interest is produced, along with its variance, using Rubin’s rules [5]. If the imputation model is appropriate, and the assumptions on the missing data mechanism hold, then the resulting estimates are unbiased with standard errors (SE) that reflect not only the variation of the data but also the uncertainty in the missing values [6].

When conducting MI, there are two general approaches that can be used to generate the imputed datasets when there is multivariate missingness: joint modelling, most commonly multivariate normal imputation (MVNI), and fully conditional specification (FCS). Under MVNI the missing covariates are assumed to jointly follow a multivariate normal distribution [7]. In contrast, FCS uses a series of univariate imputation models, one for each incomplete covariate, and imputes missing values for each variable by iterating through these models sequentially [8]. To obtain valid inferences, careful consideration must be made when constructing the imputation model such that it incorporates all features of the analysis model, in order to ensure compatibility between the imputation and analysis model [9, 10].

In simple terms, to achieve compatibility, the imputation model must at least include all the variables in the analysis model, and in the same form. It may also include additional variables, termed auxiliary variables, which can be used to improve the precision of the inference if the auxiliary variables are associated with the variables that have missing data. Auxiliary variables may additionally decrease bias if they are strong predictors of missingness [11]. In the context of a case-cohort study where the target estimand is the coefficient for the risk ratio (RR) estimated from a log-binomial model with IPW to address unequal probability of selection, two key features should be reflected in the imputation model; 1) the assumed distribution of the outcome, given the exposure and covariates (i.e. log-binomial), and 2) the weights. It is currently unclear how best to incorporate weights into MI in the context of a case-cohort analysis.

It has been previously shown that ignoring weights during MI can introduce bias into the point estimates and estimated variance produced through Rubin’s rules in the context of an IPW analysis model [12, 13]. Various approaches to incorporate weights into MI have been proposed in the literature. Previous work from Marti and Chavance [14] in a survival analysis of case-cohort data suggests that simply including the outcome, as a proxy for the weights, in the imputation model may be sufficient for incorporating the weights. In the case-cohort setting we are considering, there are only two distinct weights, representing the unequal selection for cases and controls, so the weighting variable is completely collinear with the outcome. An additional approach is to include weights and an interaction between the weights and all of the variables in the analysis model in the imputation model. Carpenter and Kenward [12] illustrated that this corrected for the bias seen when the weights are ignored in the imputation model. One difficulty with this approach is that it may be infeasible if there are several incomplete variables. Another drawback is the increased number of parameters to be estimated during imputation. Another potential approach is to use stratum-specific imputation, in which missing values for cases and non-case subcohort members are imputed separately. While many studies have compared MI approaches in the case-cohort setting [14–17], these are in the context of a time-to-event endpoint and predominantly considered MI only to address the missing exposure due to the design. Keogh [16] considered additional missingness in the covariates, but this was in the context of a survival analysis where weights were dependent on time. While there are many approaches available, it is unclear how these preform in a simple case-cohort context with missing covariate data.

The aim of this study was to compare the performance of a range of possible methods for implementing MI to handle missing covariate data in the context of a case-cohort study where the target analysis uses IPW to estimate the i) RR and ii) odds ratio (OR). Whilst the common estimand in case-cohort studies is the RR due to the ability to directly estimate this quantity without the rare-disease assumption [18], we have chosen to additionally consider the target estimand being the coefficient for the OR as this is still a commonly used estimand. The performance of the MI approaches was explored under a range of scenarios through the use of a simulation study closely based on a case study within BIS, and application of these methods to the BIS data. The ultimate goal was to provide guidance on the use of MI for handling covariate missingness in the analysis of case-cohort studies.

The paper is structured as follows. We first introduce the motivating example, a case-cohort investigation within BIS, and the target analysis models used for this study. This is followed by a description of the MI approaches to be assessed and details of a simulation study designed to evaluate these approaches based on the BIS case study. We then apply these approaches to the BIS case study. Finally, we conclude with a discussion.

## Methods

### Motivating example

The motivating example for this study comes from a case-cohort investigation within BIS [19]. A full description of BIS can be found elsewhere [2]. Briefly, it is a population-derived longitudinal birth cohort study of infants recruited during pregnancy (*n* = 1074). The research question focused on the effect of vitamin D insufficiency (VDI) at birth on the risk of food allergy at one-year. Cord blood was collected and stored after birth, and the children were followed up at one-year. During this review, the infant’s allergy status to five common food allergens (cow’s milk, peanuts, egg, sesame and cashew) was determined through a combination of a skin prick test and a food challenge. Of those who completed the one-year review (*n* = 894, 83%), a random subcohort was selected (*n* = 274), with a probability of approximately 0.31. The exposure, VDI, was defined as 25(OH)D_3_ serum metabolite levels below 50 nM and was measured from those with a confirmed food allergy at one-year and those who were selected into the subcohort.

The planned primary analysis of the case study was to estimate the RR for the target association using IPW in a binomial regression model adjusted for the confounding variables: family history of allergy (any of asthma, hay fever, eczema, or food allergy in an infant’s parent or sibling), Caucasian ethnicity of the infant, number of siblings, domestic pet ownership, and formula feeding at 6 and 12 months. The target analysis of this study adjusted for a slightly different set of confounders to the BIS example. A description of these variables and the amount of missing data in each is shown in Table 1.

**Table 1:** Detailed description of case study variables used during simulation and their distribution within the Barwon Infant Study

Variable	Variable Type	Label	n (%)^*^ (***N*** = 1074)	n (%) missing
***Outcome***				
Food Allergy at 1 year (present)	Binary; Present/Absent	*foodallergy*	61 (7.8)	288 (26.8)
***Exposure***				
Vitamin D Insufficiency at Birth (present)	Binary; Present/Absent	*vdi*	149 (44.5)	739 (68.8)
***Covariates***				
Ethnicity (Caucasian)	Binary; Caucasian/Not Caucasian	*cauc*	772 (72.1)	3 (0.3)
Maternal Vitamin D Supplements Usage (present)	Binary; Present/Absent	*antevd*	564 (78.8)	358 (33.3)
Family History of Allergy (present)	Binary; Present/Absent	*hxfamall*	911 (86.1)	16 (1.5)
Number of Siblings	3-Level Categorical	*nsib*		0 (0.00)
None			453 (42.2)	
One			383 (35.7)	
Two or more			238 (22.2)	
Family Pet Ownership (present)	Binary; Present/Absent	*petown*	815 (80.5)	62 (5.8)
Formula Feeding at 6 months^#^	3-Level Categorical	*formfeed6*		189 (17.6)
Exclusively Breast Fed			429 (46.6)	
Exclusively Formula Fed			320 (34.8)	
Mixed Feeding			171 (18.6)	
Formula Feeding at 12 months#	3-Level Categorical	*formfeed12*		154 (14.3)
Exclusively Breast Fed			271 (30.6)	
Exclusively Formula Fed			354 (40.0)	
Mixed Feeding			260 (29.4)	
***Auxiliary***				
Maternal Age at Birth		*mage*	32.1 (4.78)	3 (0.3)
Family SEIFA Classification	3-Level Categorical	*seifa*		20 (1.9)
Low			268 (25.4)	
Middle			204 (19.4)	
High			582 (55.2)	

*Mean and standard deviation given for maternal age; percentage given is exclusive of missing data

#Formula feeding variables were not included in the simulation study

*SEIFA* Socioeconomic index for area

### Target analysis

In this study, we focus on two estimands from two different analysis models. Each model targets the association between VDI and food allergy at one-year, adjusting for confounders. The first model estimates the adjusted RR using a Poisson regression model with a log-link and a robust error variance [20] to avoid the known convergence issues of the log-binomial model:

1 $${\displaystyle \begin{array}{cl}{\log} \left\{\Pr \left( \mathrm{foodallergy}=1\right)\right\}= &{\theta}_{0}+{\theta}_{1} \mathrm{vdi}+{\theta}_{2} \mathrm{cauc}+{\theta}_{3} \mathrm{petown}\\ &+{\theta}_{4}\left[ \mathrm{nsib}=1\right]+{\theta}_{5}\left[ \mathrm{nsib}=2\right]+{\theta}_{6} \mathrm{antevd}\\ &+{\theta}_{7} \mathrm{hfxamall}\end{array}}$$

The RR of interest is exp(*θ*_1_). The second target estimand is the adjusted OR for the exposure-outcome association, estimated via a logistic regression model:

2 $${\displaystyle \begin{array}{cl}\mathrm{logit}\left\{\Pr \left( \mathrm{foodallergy}=1\right)\right\}= &{\beta}_{0}+{\beta}_{1} \mathrm{vdi}+{\beta}_{2} \mathrm{cauc}+{\beta}_{3} \mathrm{petown}\\ &+{\beta}_{4}\left[ \mathrm{nsib}=1\right]+{\beta}_{5}\left[ \mathrm{nsib}=2\right]+{\beta}_{6} \mathrm{antevd}\\ &+{\beta}_{7} \mathrm{hfxamall}\end{array}}$$

The OR of interest is exp(*β*_1_). Estimation for each model uses IPW, where the weights are estimated using the method outlined by Borgan [21] for stratified sampling of the cohort, noting that the oversampling of the cases is a special case of stratified sampling where stratification depends on the outcome. The weight for *i*th individual can be defined as *w*_*i*_ = 1 for cases, and *n*_0_/*m*_0_ for non-cases, where *n*_0_ is the number of non-cases in the full cohort and *m*_0_ is the number of non-cases within the subcohort.

### MI methods

Below we outline the four approaches we considered in the BIS case study and simulation study for incorporating the weights into the imputation model. All MI approaches include the outcome, exposure, covariates, and auxiliary variables in the imputation model except where specified. Where imputation has been applied under FCS, binary variables have been imputed from a logistic model. For MVNI, all variables are imputed from a multivariate normal distribution, conditional on all other variables, with imputed covariates included into the analysis as is (i.e. without rounding).

#### Weight proxy as a main effect

Under this approach, only the analysis and auxiliary variables listed above were included in the imputation model, with the outcome acting as a proxy for the weights due to the collinearity between the outcome and weights. This approach is implemented under both the FCS and MVNI frameworks.

#### Weight proxy interactions

The second approach includes two-way interactions between the outcome (as a proxy for the weights) and all other analysis variables in the imputation model. Within FCS, the interactions were included as predictors, with these derived within each iteration of the imputation [22]. Within MVNI interactions were considered as ‘just another variable’ in the imputation model [22].

#### Stratum-specific imputation

In the case-cohort setting, where there are only two weight strata, another option is to impute separately within each weight/outcome stratum. Here, the outcome is not included in the imputation model, but rather the incomplete covariates are imputed using a model including the exposure, other covariates and auxiliary variables, for cases and non-cases separately.

#### Weighted imputation model

A final option is to impute the missing values using a weighted imputation model, where the weights are set to those used during analysis. This can only be conducted within the FCS framework.

The approaches for handling the missing covariate data are summarised in Table 2.

**Table 2:** Description of multiple imputation approaches considered to handle missing covariate data

Method*	Accommodation of Weighting in MI	MI Framework	Label
Complete case	No imputation completed. Analysis applied to observations with complete covariate data.	N/A	*CCA*
Weight only	Imputation models include weights (through the outcome) as a predictor of missingness	FCS	*FCS-WO*
		MVNI	*MVNI-WO*
Weight interactions	Interaction between outcome (proxy for weight) and exposure/covariates included in imputation model through passive imputation (FCS) or ‘just another variable’ (MVNI), in addition to outcome as a predictor.	FCS	*FCS-WX*
		MVNI	*MVNI-WX*
Stratum specific imputation	Covariates imputed separately by weight status	FCS	*FCS-SS*
		MVNI	*MVNI-SS*
Weighted model	Imputation model weighted with inverse probability of selection, outcome included as a predictor.	FCS	*FCS-WM*

*All methods involve using multiple imputation to address the missing covariates, excluding the complete case analysis, with a weighted analysis model to address the unequal probabilities and missing exposure

*FCS* Fully Conditional Specification, *MVNI* Multivariate Normal Imputation, *MI* Multiple Imputation

### Simulation study

A simulation study was conducted to assess the performance of each MI approach for accommodating the case-cohort weights into the imputation model, across a range of scenarios. Simulations were conducted using Stata 15.1 [23]. Cohorts of size 1000 were generated using models outlined above with parameter values based on the observed relationships in BIS (except where noted).

#### Complete data generation

**Fig. 1:**
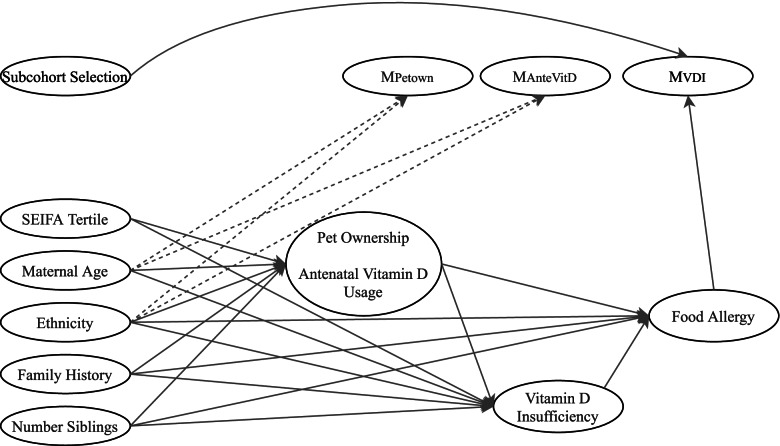
Missingness directed acyclic graph (m-DAG) depicting the assumed causal structure between simulated variables and missingness indicators under the dependent missing mechanisms. For the independent missing mechanism, the dashed lines are absent. For simplicity, associations between baseline covariates have not been shown

Complete data, comprising the exposure, five confounders and two auxiliary variables, were generated sequentially using the models listed below. Models for data generation were constructed based on the plausible causal structure specified in Fig. 1. A table showing the parameter values can be found in Additional file 1.

i. Caucasian ethnicity

3 $$\mathrm{cauc}\sim \mathrm{Bernoulli}\left(\mathrm{p}\right)$$

ii. Maternal age at birth, in years, (auxiliary variable)

4 $${\displaystyle \begin{array}{c}\mathrm{mage}={\delta}_0+{\delta}_1\mathrm{cauc}+\epsilon \end{array}}$$

where ε~*N*(0, *σ*^2^)

iii. Socioeconomic Index for Areas (SEIFA) tertile, (auxiliary variable)

5 $${\displaystyle \begin{array}{c}\log \left\{\frac{\Pr \left(\mathrm{seifa}=1\right)}{\Pr \left(\mathrm{seifa}=0\right)}\right\}={\zeta}_0+{\zeta}_1\mathrm{mage}+{\zeta}_2\mathrm{cauc}\end{array}}$$

6 $${\displaystyle \begin{array}{c}\log \left\{\frac{\Pr \left(\mathrm{seifa}=2\right)}{\Pr \left(\mathrm{seifa}=0\right)}\right\}={\eta}_0+{\eta}_1\mathrm{mage}+{\eta}_2\mathrm{cauc}\end{array}}$$

iv. Family history of allergy

7 $${\displaystyle \begin{array}{c}\mathrm{logit}\left\{\Pr \left(\mathrm{hxfamall}=1\right)\right\}={\iota}_0+{\iota}_1\mathrm{cauc}\end{array}}$$

  v. Number of siblings

8 $${\displaystyle \begin{array}{cl}\log \left\{\frac{\Pr \left( \mathrm{nsib}=1\right)}{\Pr \left( \mathrm{nsib}=0\right)}\right\}= & {\kappa}_{0}+{\kappa}_{1} \mathrm{mage}+{\kappa}_{2} \mathrm{cauc} + {\kappa}_{3}\left[\mathrm{seifa}=1\right]\\ &+{\kappa}_{4}\left[\mathrm{seifa}=2\right]+{\kappa}_{5}\mathrm{hxfamall}\end{array}}$$

9 $${\displaystyle \begin{array}{cl}\log \left\{\frac{\Pr \left( \mathrm{nsib}=2\right)}{\Pr \left( \mathrm{nsib}=0\right)}\right\}= & {\lambda}_{0}+{\lambda}_{1} \mathrm{mage}+{\lambda}_{2} \mathrm{cauc} + {\lambda}_{3}\left[\mathrm{seifa}=1\right]\\ &+{\lambda}_{4}\left[\mathrm{seifa}=2\right]+{\lambda}_{5}\mathrm{hxfamall}\end{array}}$$

vi. Pet ownership and antenatal vitamin D supplement usage

10 $${\displaystyle \begin{array}{cl}\mathrm{logit}\left\{\Pr\left( \mathrm{petown}=1\right)\right\}= & {\rho}_{0}+{\rho}_{1} \mathrm{mage} +{\rho}_{2} \mathrm{cauc} + {\rho}_{3}\left[ \mathrm{seifa}=1\right]\\ &+{\rho}_{4}\left[ \mathrm{seifa}=2\right]+{\rho}_{5}\mathrm{hxfamall}\\ &+{\rho}_{6}\left[ \mathrm{nsib}=1\right]+{\rho}_{7}\left[ \mathrm{nsib}=2\right]\end{array}}$$

11 $${\displaystyle \begin{array}{cl}\mathrm{logit}\left\{\Pr\left( \mathrm{antevd}=1\right)\right\}= & {\phi}_{0}+{\phi}_{1} \mathrm{mage} + {\phi}_{2} \mathrm{cauc}+ {\phi}_{3}\left[ \mathrm{seifa}=1\right]\\ &+{\phi}_{4}\left[ \mathrm{seifa}=2\right]+{\phi}_{5}\mathrm{hxfamall}\\ & +{\phi}_{6}\left[ \mathrm{nsib}=1\right]+{\phi}_{7}\left[ \mathrm{nsib}=2\right]\end{array}}$$

vii. The exposure, VDI

12 $${\displaystyle \begin{array}{cl}\mathrm{logit}\left\{\Pr\left( \mathrm{vdi}=1\right)\right\}= & {\psi}_0+{\psi}_{1} \mathrm{mage} + {\psi}_{2} \mathrm{cauc}+ {\psi}_{3}\left[ \mathrm{seifa}=1\right]\\ &+{\psi}_{4}\left[ \mathrm{seifa}=2\right]+{\psi}_{5}\mathrm{hxfamall} +{\psi}_{6}\left[ \mathrm{nsib}=1\right]\\ &+{\psi}_{7}\left[ \mathrm{nsib}=2\right]+{\psi}_{8} \mathrm{petown} + {\psi}_{9} \mathrm{antevd}\end{array}}$$

Finally, the outcome, food allergy at one-year, was generated from a Bernoulli distribution with a probability determined by either model (1) or model (2) so the target analysis was correctly specified. In these models we set *θ*_1_ = log(*RR*_*adj*_) = log(1.16) and *β*_1_ = log(*OR*_*adj*_) = log(1.18) as estimated from BIS. Given the weak exposure-outcome association in BIS, we also generated food allergy with an enhanced association where we set *θ*_1_ = *β*_1_ = log(2.0).

An additional extreme data generation scenario was considered as a means to stress-test the MI approaches under more extreme conditions. In this scenario, the associations between the continuous auxiliary variable of maternal age and the exposure, missing covariates, and missing indicator variables were strengthened.

#### Inducing Missingness

Missingness was introduced into two covariates, antenatal vitamin D usage and pet ownership. Missingness was generated such that *p*% of overall observations had incomplete covariate information, with $\frac{p}{2}\%$ having missing data in just one covariate and $\frac{p}{2}\%$ having missing data in both, where *p* was chosen as either 15 or 30. Three missing data mechanisms were considered: an independent missing data mechanism and two dependent missing data mechanisms as depicted in the missingness directed acyclic graph (m-DAG) in Fig. 1.

Under the independent missing data mechanism, missingness in each covariate was randomly assigned to align with the desired proportions. Under the dependent missingness mechanisms, an indicator for missingness in pet ownership, M_petown_, was initially generated from a logistic model (13), followed by an indicator for missingness in antenatal vitamin D usage, M_antevd_ (model (14)).

13 $${\displaystyle \begin{array}{c}\mathrm{logit}\left\{\Pr \left({\mathrm{M}}_{\mathrm{petown}}=1\right)\right\}={\nu}_0+{\nu}_1\mathrm{foodallergy}+{\nu}_2\mathrm{cauc}+{\nu}_3\mathrm{mage}\end{array}}$$

14 $${\displaystyle \begin{array}{c}\mathrm{logit}\left\{\Pr \left({\mathrm{M}}_{\mathrm{antevd}}=1\right)\right\}={\tau}_0+{\tau}_1\mathrm{foodallergy}+{\tau}_2\mathrm{cauc}+{\tau}_3\mathrm{mage}+{\tau}_4{\mathrm{M}}_{\mathrm{petown}}\end{array}}$$

The parameters, *ν*_0_ and *τ*_0_, and *τ*_4_ were iteratively chosen until the desired proportions of missing information were obtained. Missingness indicators were generated dependent on the outcome (a setting where the complete-case analysis would be expected to be biased) and an auxiliary variable (a setting where we expect a benefit of MI over the complete-case analysis), as depicted in the causal diagram in Fig. 1. The dependency between the missing indicator variables was used to simultaneously control both the overall proportion of incomplete records and the proportion with missingness in both variables.

The two dependent missing mechanisms differed in the strength of association between predictor variables and the missing indicators. The first mechanism used parameter values set to those estimated in BIS (termed Dependent Missingness – Observed, or *DMO*). The second used an enhanced mechanism where the parameters values were doubled (termed Dependent Missingness – Enhanced, or *DME*). The parameter values used to induce missingness under the dependent missingness mechanisms can be found in Additional file 1.

To mimic the case-cohort design, a subcohort was then randomly selected using one of three probabilities of selection (0.20, 0.30, 0.40). The exposure, VDI, was set to missing for participants without the outcome and who had not been selected into the subcohort.

Overall, we considered 78 scenarios (2 data generation processes, 2 exposure-outcome associations, 3 missing data mechanisms, 2 incomplete covariate proportions, and 3 subcohort selection probabilities, plus another 6 scenarios under extreme conditions).

For the 6 extreme scenarios presented in the results section, the mean percentage of cases in the full cohort across the 2000 simulated datasets was 20.4% (standard deviation: 1.3%) for scenarios targeting RR estimation, and 18.4% (1.2%) for OR estimation. The average case-cohort sample size ranged from 348 to 522, increasing with the probability of subcohort selection. The percentage of incomplete observations within the case-cohort sample ranged from 30.5 to 32.6%, with the percentage of incomplete cases increasing as the subcohort size decreased due to the dependency between the probability of being incomplete and the outcome. Additional file 1 contains a table showing summaries for the 2000 simulated datasets for the 6 extreme scenarios.

#### Evaluation of MI approaches

For each scenario, the MI approaches outlined above were applied and 30 imputed datasets generated, to match the maximum proportion of missing observations. The imputed datasets were analysed using IPW with the corresponding target analysis model. A complete-data analysis (with no missing data in the subcohort) and a complete-case analysis (CCA) were also conducted for comparison. Performance was measured in terms of percentage bias relative to the true value (relative bias), the empirical and model-based SE, and coverage probability of the 95% confidence interval for the target estimand, the effect of VDI on food allergy (*θ*_1_ in model (1) and *β*_1_ in model (2)). In calculating the performance measures, the true value was taken to be the value used during data generation with measures calculated using the simsum package in Stata (see [24] for details). We also report the Monte Carlo standard error (MCSE) for each performance measure. For each scenario we presented results for 2000 simulations. With 2000 simulations, the MCSE for a true coverage of 95% would be 0.49%, and we can expect the estimated coverage probability to fall between 94.0 and 96.0% [25]. Since convergence issues were expected across the methods, we generated 2200 datasets in each scenario and retained the first 2000 datasets on which all methods converged. These 2000 datasets were used to calculate all performance measures except the convergence rate, which was calculated across the 2200 simulations.

#### Bias in RR estimation

Incompatibility between the imputation and analysis model may arise due to the imputation of missing values from a linear or logistic model when the analysis targets the RR [26]. To explore the bias introduced into the point estimate in this context, MI was conducted on the full cohort with completely observed exposure (i.e. before data were set to missing by design) and analysed without weighting. This was conducted to understand the baseline level bias, prior to the introduction of weighting. Results for this analysis are presented in Additional file 2.

### Case study

Each of the MI methods were also applied to the target analyses using BIS data. For consistency with the simulation study, the analysis was limited to observations with complete outcome and exposure data (*n* = 246). In the case study there were also missing values in the covariates, Caucasian ethnicity (1%) and family history of allergy (1%), and the auxiliary variable SEIFA tertiles (2%), which were imputed alongside pet ownership (1%) and antenatal vitamin D usage (23%). For the FCS approaches, all variables were imputed using a logistic model, except for SEIFA tertile, which was imputed using an ordinal logistic model. Imputed datasets were analysed under each target analysis model with weights of 1 for cases and (0.31)^−1^ for non-case subcohort members. A CCA was also conducted.

## Results

Given that the pattern of results were similar across the range of scenarios, we describe the results for the 6 scenarios under extreme conditions (enhanced exposure-outcome association, 30% missing covariates under *DME,* and enhanced auxiliary associations). The results for the remaining scenarios are provided in Additional file 3.

Across the 2200 simulations, only FCS-WX and FCS-SS had convergence issues (i.e., successfully completing the analysis without non-convergence of the imputation procedure or numerical issues in the estimation). The rate of non-convergence was greatest for FCS-WX with the smallest subcohort size (probability of selection = 0.2), with 4.0% of simulations under RR estimation and 3.2% under OR estimation failing to converge. Less than 0.2% of simulations failed to converge for FCS-SS under any combination of estimand and subcohort probability of selection.

**Fig. 2:**
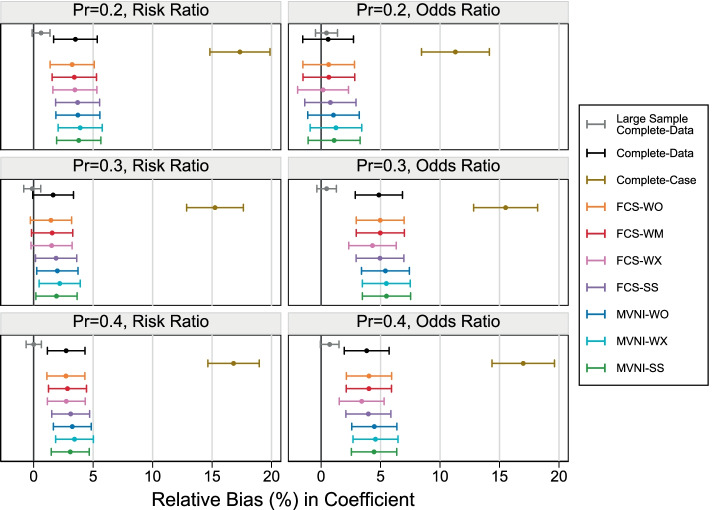
Relative bias in the coefficient under the extreme scenarios with 30% missing covariate information. Error bars represent 1.96xMonte Carlo standard errors

Figure 2 shows the relative bias in the estimate of the association (RR or OR) between VDI and food allergy at one-year for each scenario considered under extreme conditions. The largest bias for the large sample complete-data analysis occurred for the coefficient of the OR and the largest subcohort size at 1.5%, with the MCSE range covering a relative bias of 0%. In all scenarios shown, the CCA resulted in a large relative bias, ranging between 10 and 20%. All MI approaches reduced this bias drastically, irrespective of estimand and subcohort size. When the target estimand was the coefficient for the OR and the smallest subcohort probability was used, all MI approaches were approximately unbiased, with MVNI-WX showing the largest relative bias at 1.4% and FCS-WX showing the least at 0.4%. For the remaining scenarios across both estimands, the complete-data analysis and MI approaches showed a positive bias, with the largest occurring with the log (OR) estimand and a probability of selection of 0.3, where the relative bias was centred around 5%. Overall, minimal differences can be seen between the MI approaches when the estimation targeted the RR. When the target estimand was the log (OR), there was a slight decrease in relative bias for FCS-WX, when compared to other MI approaches, and a slight increase in the relative bias for MVNI approaches, when compared to FCS approaches.

**Fig. 3:**
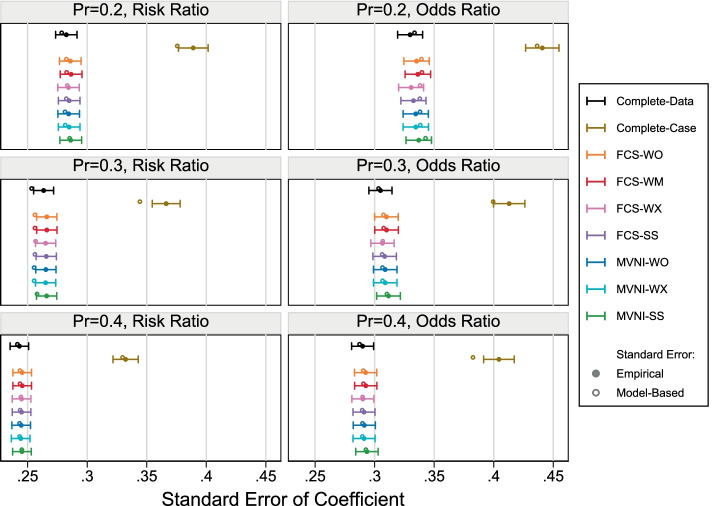
Empirical standard error and model based standard error under the extreme scenarios with 30% missing covariate information. Error bars represent 1.96xMonte Carlo standard errors

The empirical SE and model-based SE are shown in Fig. 3. For most scenarios, we can see the SE has been underestimated in the CCA. There was a slight underestimation of the SE when the subcohort selection probability was 0.3 and the target estimand the log (RR), however, the model-based SE appears to fall within the MCSE intervals for the empirical SE. There appears to be no systematic deviation between the empirical SE and the model-based SE for any scenario. An increase in the precision can be seen as the subcohort size increases, and there is an increase in precision for all MI methods compared to the CCA for any given scenario, as expected.

**Fig. 4:**
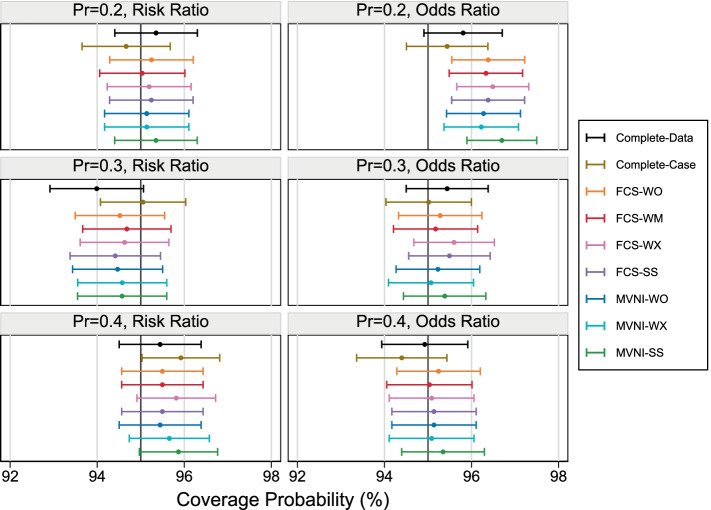
Coverage probability across 2000 simulations under the extreme scenarios with 30% missing covariate information. Error bars represent 1.96xMonte Carlo standard errors

The estimated coverage probabilities are shown in Fig. 4. For a nominal coverage of 95%, all MI approaches have a satisfactory coverage with 95% falling within the MCSE range for all scenarios, with the exception of the smallest subcohort size with OR estimation. Under this scenario, all MI approaches produce over-coverage, as a result of the point estimate being unbiased and the SE overestimated (but with the average model-based SE falling within the MCSE range). There is no apparent pattern in the coverage probability across the MI methods, with all methods performing similarly. Results from the CCA showed acceptable levels of coverage.

**Fig. 5:**
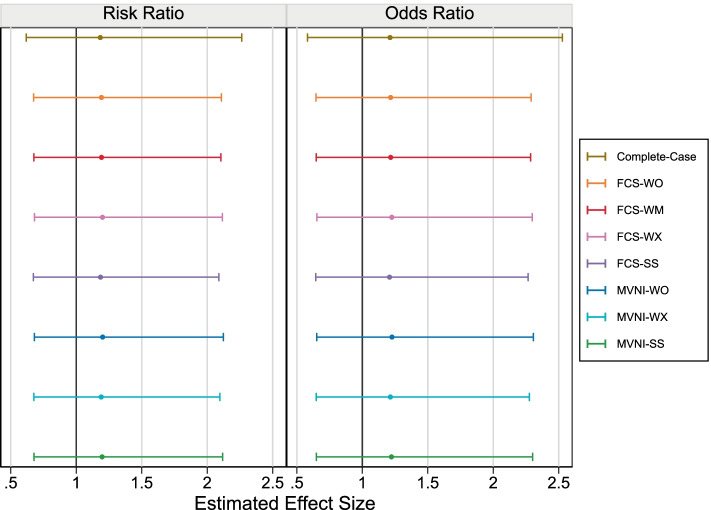
Estimated parameter value with 95% confidence interval in case study dataset

The results from the case study are shown in Fig. 5. Results are consistent with the simulation study in that there is little variation in the estimated association across the MI methods. Unlike the simulation study under extreme conditions, the estimated coefficient is similar in the CCA and the MI approaches. There is an expected recovery of information leading to an increase in precision for MI approaches compared to the CCA.

## Discussion

In this study we compared a number of different approaches for accommodating unequal sampling probabilities into MI in the context of a case-cohort study. We found that how the weights were included in the imputation model had minimal effect on the estimated association or performance of MI which, as expected, outperformed CCA. Results were consistent across different levels of missing covariate information, target estimand and subcohort selection probability.

While bias was seen in some scenarios, this was minimal (~ 5%) and consistent across all MI approaches. We conducted a large sample analysis to confirm the data generation process was correct, given the bias observed in the complete-data analysis, which showed minimal bias. We have therefore attributed the positive bias seen in the simulation study to a finite sampling bias, which was observed for large effect sizes in similar studies [16]. The minimal difference across MI methods seen in the current study may be due to the case-cohort setting having only two weight strata that are collinear with the outcome and all MI approaches including the outcome either directly or indirectly (in the case of stratum-specific imputation). The results of this study complement the work by Marti and Chavance [14] who showed that inclusion of the outcome in the imputation model was sufficient to account for the unequal sampling probabilities in the context of a case-cohort survival analysis. In the case study, the MI approaches performed similarly to the CCA and we believe this is due to the observed weak associations in BIS.

Our simulation study was complicated by potential bias due to the incompatibility between the imputation and analysis model when the target analysis estimated a RR through a Poisson regression model. The same would be true if the RR was estimated using a binomial model, as in the case study. We assessed this explicitly through imputation of the full cohort prior to subcohort selection, with results shown in Additional file 2. Minimal bias was seen due to this incompatibility. This may be because we only considered missing values in the covariates, which have been generated from a logistic model. Studies that have shown bias due to this incompatibility had considered missing values in both the outcome and exposure [26].

One strength of the current study was that it was based on a real case study, with data generated under a causal structure depicted by m-DAGs informed by subject matter knowledge. This simulation study also examined a range of scenarios; however, it is important to note that not all possible scenarios can be considered, and these results may not extend to scenarios with missingness dependent on unobserved data or with unintended missingness in the exposure or outcome. This study also has a number of limitations. The simulations were conducted under controlled conditions such that the analysis model was correctly specified, and the missing data mechanism was known. Under the specified missing data mechanisms, the estimand was known to be recoverable and therefore MI was expected to perform well [27]. The missing data mechanism is generally unknown in a real data setting and results may not be generalisable.

Another limitation of this study is that only covariates have been considered incomplete. Often there can be missingness in the outcome (e.g. subcohort members drop-out prior to one-year follow-up and outcome collection) and/or unintended missingness in the exposure (e.g. cord blood not stored for infants selected into the subcohort or with food allergy). This study has also only considered a combination of MI and IPW. There are other analytic approaches that could have been used, for example using weighting to account for the missing covariates as well as the missing data by design, or imputing the exposure in those not in the subcohort and conducting a full cohort analysis. These approaches have been explored in a time-to-event setting [14, 16, 17] but little is known on the appropriateness for the case-cohort setting with a binary outcome. Furthermore, no study to date has considered the scenario of additional exposure missing by chance within the subcohort. The limitations mentioned here offer an avenue for future work.

## Conclusions

When performing MI in the context of case-cohort studies, how unequal sampling probabilities were accounted for in the imputation model made minimal difference in the analysis. In this setting, inclusion of the outcome in the imputation model, which is already standard practice, was a sufficient approach to account for the unequal sampling probabilities incorporated in the analysis model.

## Supplementary Information

**Additional file 1.**

**Additional file 2.**

**Additional file 3.**

## Data Availability

The datasets used and/or analysed during the current study are available from the corresponding author on reasonable request.

## Abbreviations

BIS: Barwon Infant Study
CCA: Complete-Case Analysis
DME: Dependent Missing – Enhanced
DMO: Dependent Missing – Observed
FCS: Fully Conditional Specification
IPW: Inverse Probability Weighting
m-DAG: Missingness Directed Acyclic Graph
MCSE: Monte Carlo Standard Error
MI: Multiple Imputation
MVNI: Multivariate Normal Imputation
OR: Odds Ratio
RR: Risk Ratio
SE: Standard Error
SEIFA: Socioeconomic Index for Areas
VDI: Vitamin D Insufficiency
