# Supplementary material for: Evaluation of multiple imputation approaches for handling missing covariate information in a case-cohort study with a binary outcome
Source: BMC Med Res Methodol. 2022 Apr 3;22:87. doi: 10.1186/s12874-021-01495-4 (PMC8978363; doi:10.1186/s12874-021-01495-4)
Supplement: Supplementary file 1 — Additional file 1. [file 12874_2021_1495_MOESM1_ESM.docx]

# **Additional file 1: Simulation Study Data Generation**

[**Supplementary Table 1:** Exponentiated parameter values used during complete data generation 2](#_Toc91851095)

[**Supplementary Table 2:** Fixed parameter values used to generate missing indicators for covariates under the dependent missing mechanisms. 3](#_Toc91851096)

[**Supplementary Table 3:** Descriptive statistics for the 2000 simulated datasets under each of the extreme scenarios 3](#_Toc91851097)

Additional file 1 contains supplementary material supporting the data generation of the simulation study.

- Table S1 provides parameter values used in the data generation models, with the exponentiated value given for multinomial, logistic and Poisson regression models. Parameter values were obtained from fitting the data generation models (equations 1-12) to the case study data from the Barwon Infant Study, and where a data generation scenario has required an enhanced association, this has been indicated.
- Table S2 provides the exponentiated parameter values corresponding to each predictor of missingness used in the dependent missing data generation models. Under the “observed dependent missing” scenarios, parameter values were obtained by fitting the logistic regression models to the case study data (equations 13-14).
- Table S3 provides the mean and standard deviation for the outcome prevalence, case-cohort sample size, and percentage of incomplete cases within the case-cohort sample, across the 2000 simulated datasets for each of the 6 scenarios under extreme conditions.

**Supplementary Table 1:** Exponentiated parameter values used during complete data generation

| **Dependent Variable** | | **Model** | **Data Generation Scenario** | **Base Odds*** | **Independent Variable** | | | | | | | | | |
| --- | --- | --- | --- | --- | --- | --- | --- | --- | --- | --- | --- | --- | --- | --- |
|  |  |  |  |  | *cauc* | *mage* | *seifa=1* | *seifa=2* | *hxfamall* | *nsib=1* | *nsib=2* | *petown* | *antevd* | *vdi* |
| *Cauc* | | Bernouli | All | 0.72 |  |  |  |  |  |  |  |  |  |  |
|  | |  |  |  |  |  |  |  |  |  |  |  |  |  |
| *mage* | | Linear# | All | 31.33 | 1.01 |  |  |  |  |  |  |  |  |  |
|  | |  |  |  |  |  |  |  |  |  |  |  |  |  |
| *Seifa* | |  |  |  |  |  |  |  |  |  |  |  |  |  |
|  | *seifa = 1* | Multinomial | All | 0.10 | 1.65 | 1.05 |  |  |  |  |  |  |  |  |
|  | *seifa = 2* |  |  | 0.17 | 1.17 | 1.08 |  |  |  |  |  |  |  |  |
|  |  |  |  |  |  |  |  |  |  |  |  |  |  |  |
| *Hxfamall* | | Logistic | All | 4.94 | 1.38 |  |  |  |  |  |  |  |  |  |
|  | |  |  |  |  |  |  |  |  |  |  |  |  |  |
| *Nsib* | |  |  |  |  |  |  |  |  |  |  |  |  |  |
|  | *nsib = 1* | Multinomial | All | 0.02 | 0.81 | 1.11 | 0.74 | 0.92 | 2.29 |  |  |  |  |  |
|  | *nsib = 2* |  |  | 0.0004 | 0.77 | 1.21 | 1.43 | 1.11 | 3.39 |  |  |  |  |  |
|  |  |  |  |  |  |  |  |  |  |  |  |  |  |  |
| *Petown* | | Logistic | Observed/Enhanced | 11.84 | 1.17 | 0.97 | 1.33 | 0.74 | 0.83 | 1.11 | 1.36 |  |  |  |
|  |  |  | Extreme |  |  | 0.90 |  |  |  |  |  |  |  |  |
|  | |  |  |  |  |  |  |  |  |  |  |  |  |  |
| *Antevd* | | Logistic | All | 0.03 | 1.36 | 1.16 | 1.11 | 0.98 | 1.49 | 0.51 | 0.28 |  |  |  |
|  | |  |  |  |  |  |  |  |  |  |  |  |  |  |
| *vdi* | | Logistic | Observed/Enhanced | 0.86 | 0.88 | 1.04 | 1.40 | 1.01 | 0.34 | 0.71 | 1.01 | 0.98 | 0.51 |  |
|  |  |  | Extreme |  |  | 1.11 |  |  |  |  |  |  |  |  |
|  | |  |  |  |  |  |  |  |  |  |  |  |  |  |
| *foodallergy* | | Poisson | Observed | 0.07 | 1.08 |  |  |  | 1.93 | 1.42 | 1.38 | 0.31 | 0.80 | 1.16 |
|  |  |  | Enhanced/Extreme |  |  |  |  |  |  |  |  |  |  | 2.00 |
|  |  |  |  |  |  |  |  |  |  |  |  |  |  |  |
|  |  | Logistic | Observed | 0.08 | 1.08 |  |  |  | 2.02 | 1.48 | 1.43 | 0.27 | 0.78 | 1.18 |
|  |  |  | Enhanced/Extreme |  |  |  |  |  |  |  |  |  |  | 2.00 |

*Probability given for Bernoulli models, intercept for linear models and base risk for Poisson model
#Error terms were generated with a standard deviation of 4

**Supplementary Table 2:** Fixed parameter values used to generate missing indicators for covariates under the dependent missing mechanisms.

| Independent Variable |  | Missing Indicator Variable | | | | | | |
| --- | --- | --- | --- | --- | --- | --- | --- | --- |
|  |  | $M_{petown}$ | | |  | $M_{antevitd}$ | | |
|  |  | Dependent Missing - Observed | Dependent Missing - Enhanced | Extreme Scenario |  | Dependent Missing - Observed | Dependent Missing - Enhanced | Extreme Scenario |
| *foodallergy* |  | 1.83 | 3.35 | 3.35 |  | 0.79 | 0.62 | 0.62 |
| *cauc* |  | 0.66 | 0.44 | 0.44 |  | 0.74 | 0.55 | 0.55 |
| *mage* |  | 0.89 | 0.79 | 0.79 |  | 0.96 | 0.92 | 0.90 |

Values are given in the exponentiated form and represent adjusted odds ratios.

**Supplementary Table 3:** Descriptive statistics for the 2000 simulated datasets under each of the extreme scenarios

| **Subcohort selection probability** | **Descriptive** |  | **Modified Poisson Model** | |  | **Logistic Regression** | |
| --- | --- | --- | --- | --- | --- | --- | --- |
|  |  |  | Mean | SD |  | Mean | SD |
| 0.2 | Percent outcome |  | 20.4% | 1.30% |  | 18.40% | 1.20% |
|  | Case-cohort sample size |  | 363 | 15.4 |  | 348 | 15.1 |
|  | Percent incomplete in case-cohort |  | 32.6% | 2.3% |  | 32.6% | 2.3% |
|  |  |  |  |  |  |  |  |
| 0.3 | Percent outcome |  | 20.4% | 1.3% |  | 18.4% | 1.2% |
|  | Case-cohort sample size |  | 443 | 15.5 |  | 429 | 15.9 |
|  | Percent incomplete in case-cohort |  | 31.3% | 1.9% |  | 31.3% | 1.9% |
|  |  |  |  |  |  |  |  |
| 0.4 | Percent outcome |  | 20.4% | 1.3% |  | 18.4% | 1.2% |
|  | Case-cohort sample size |  | 522 | 15.7 |  | 510 | 15.5 |
|  | Percent incomplete in case-cohort |  | 30.5% | 1.8% |  | 30.5% | 1.8% |

*SD: Standard deviation*
