# Supplementary material for: Evaluation of multiple imputation approaches for handling missing covariate information in a case-cohort study with a binary outcome
Source: BMC Med Res Methodol. 2022 Apr 3;22:87. doi: 10.1186/s12874-021-01495-4 (PMC8978363; doi:10.1186/s12874-021-01495-4)
Supplement: Supplementary file 2 — Additional file 2. [file 12874_2021_1495_MOESM2_ESM.docx]

# **Additional file 2: Bias due to incompatible imputation model when estimating a risk ratio results**

[**Supplementary Table 4:** Bias (relative bias %) in the coefficient for the association between vitamin D insufficiency and food allergy following imputation in the full cohort, for an observed exposure-outcome association. 2](#_Toc91851069)

[**Supplementary Table 5:** Bias (relative bias %) in the coefficient for the association between vitamin D insufficiency and food allergy following imputation in the full cohort, for an enhanced exposure-outcome association. 3](#_Toc91851070)

Additional file 2 provides supplementary material for the additional analyses assessing the bias in risk ratio estimation following multiple imputation from an incompatible model.

- Table S4 provides the bias and relative bias for 12 scenarios (2x missing proportion, 2x estimand, and 3x missing data mechanism) under the observed exposure-outcome association, where multiple imputation and analysis has been conducted in the full cohort (n=1,000) prior to case-cohort sample selection.
- Table S5 provides the same measures for the 12 scenarios under the enhanced exposure-outcome association.

**Supplementary Table 4:** Bias (relative bias %) in the coefficient for the association between vitamin D insufficiency and food allergy following imputation in the full cohort, for an observed exposure-outcome association.

| **Missing data mechanism** | **Method** |  | **Modified Poisson Model** | | | |  | **Logistic Model** | | | |
| --- | --- | --- | --- | --- | --- | --- | --- | --- | --- | --- | --- |
|  |  |  | **15% Missing** | | **30% Missing** | |  | **15% Missing** | | **30% Missing** | |
| Independent | *Complete Data* |  | -0.01 | (-9.03) | 0.00 | (-3.13) |  | -0.01 | (-4.53) | 0.00 | (2.78) |
|  | *MI-FCS* |  | -0.01 | (-9.47) | -0.01 | (-3.77) |  | -0.01 | (-4.60) | 0.00 | (2.33) |
|  | *MI-MVNI* |  | -0.01 | (-9.39) | -0.01 | (-3.51) |  | -0.01 | (-4.45) | 0.00 | (2.62) |
| *MC standard errors range* | |  | 0.01 - 0.01 | | 0.01 - 0.01 | |  | 0.01 - 0.01 | | 0.01 - 0.01 | |
|  |  |  |  |  |  |  |  |  |  |  |  |
| Dependent - Observed | *Complete Data* |  | 0.00 | (-3.06) | 0.01 | (5.28) |  | 0.00 | (-0.64) | 0.00 | (-1.75) |
|  | *MI-FCS* |  | 0.00 | (-3.33) | 0.01 | (4.89) |  | 0.00 | (-0.87) | 0.00 | (-2.35) |
|  | *MI-MVNI* |  | 0.00 | (-3.07) | 0.01 | (4.96) |  | 0.00 | (-0.55) | 0.00 | (-1.76) |
| *MC standard errors range* | |  | 0.01 - 0.01 | | 0.01 - 0.01 | |  | 0.01 - 0.01 | | 0.01 - 0.01 | |
|  |  |  |  |  |  |  |  |  |  |  |  |
| Dependent - Enhanced | *Complete Data* |  | 0.00 | (-1.15) | -0.01 | (-9.56) |  | 0.00 | (2.49) | 0.01 | (4.13) |
|  | *MI-FCS* |  | 0.00 | (-1.39) | -0.01 | (-9.77) |  | 0.01 | (3.08) | 0.01 | (4.29) |
|  | *MI-MVNI* |  | 0.00 | (-0.99) | -0.01 | (-9.55) |  | 0.01 | (3.50) | 0.01 | (4.79) |
| *MC standard errors range* | |  | 0.01 - 0.01 | | 0.01 - 0.01 | |  | 0.01 - 0.01 | | 0.01 - 0.01 | |

*Relative bias is the percentage bias relative to the true value used during data generation*

**Supplementary Table 5:** Bias (relative bias %) in the coefficient for the association between vitamin D insufficiency and food allergy following imputation in the full cohort, for an enhanced exposure-outcome association.

| **Missing Data Mechanism** | **Method** |  | **Modified Poisson Model** | | | |  | **Logistic Model** | | | |
| --- | --- | --- | --- | --- | --- | --- | --- | --- | --- | --- | --- |
|  |  |  | **15% Missing** | | **30% Missing** | |  | **15% Missing** | | **30% Missing** | |
| Independent | *Complete Data* |  | 0.00 | (0.36) | 0.01 | (0.87) |  | 0.02 | (2.23) | 0.01 | (1.35) |
|  | *MI-FCS* |  | 0.00 | (0.41) | 0.00 | (0.61) |  | 0.02 | (2.27) | 0.01 | (1.28) |
|  | *MI-MVNI* |  | 0.00 | (0.37) | 0.00 | (0.51) |  | 0.02 | (2.29) | 0.01 | (1.33) |
| *MC standard errors range* | |  | 0 – 0 | | 0 – 0 | |  | .01 - .01 | | 0.01 - 0.01 | |
|  |  |  |  |  |  |  |  |  |  |  |  |
| Dependent - Observed | *Complete Data* |  | 0.00 | (0.37) | 0.00 | (-0.67) |  | 0.01 | (2.16) | 0.00 | (0.34) |
|  | *MI-FCS* |  | 0.00 | (0.29) | -0.01 | (-0.85) |  | 0.02 | (2.22) | 0.00 | (0.71) |
|  | *MI-MVNI* |  | 0.00 | (0.3) | -0.01 | (-0.88) |  | 0.02 | (2.31) | 0.01 | (0.84) |
| *MC standard errors range* | |  | 0 – 0 | | 0 – 0 | |  | .01 - .01 | | 0.01 - 0.01 | |
|  |  |  |  |  |  |  |  |  |  |  |  |
| Dependent - Enhanced | *Complete Data* |  | 0.00 | (-0.63) | 0.00 | (0.43) |  | 0.01 | (0.86) | 0.01 | (2.08) |
|  | *MI-FCS* |  | 0.00 | (-0.56) | 0.00 | (0.46) |  | 0.01 | (0.76) | 0.01 | (2.08) |
|  | *MI-MVNI* |  | 0.00 | (-0.48) | 0.00 | (0.46) |  | 0.01 | (0.94) | 0.02 | (2.30) |
| *MC standard errors range* | |  | 0 - 0 | | 0 – 0 | |  | 0.01 - 0.01 | | 0.01 - 0.01 | |
|  |  |  |  |  |  |  |  |  |  |  |  |
| Extreme | *Complete Data* |  |  |  | 0.03 | (4.07) |  |  |  | 0.01 | (1.40) |
|  | *MI-FCS* |  |  |  | 0.03 | (4.03) |  |  |  | 0.01 | (1.26) |
|  | *MI-MVNI* |  |  |  | 0.03 | (4.67) |  |  |  | 0.01 | (1.67) |
| *MC standard errors range* | |  |  |  | 0.01 - 0.01 | |  |  |  | 0.01 - 0.01 | |

*Relative bias is the percentage bias relative to the true value used during data generation*
