# Supplementary material for: Evaluation of multiple imputation approaches for handling missing covariate information in a case-cohort study with a binary outcome
Source: BMC Med Res Methodol. 2022 Apr 3;22:87. doi: 10.1186/s12874-021-01495-4 (PMC8978363; doi:10.1186/s12874-021-01495-4)
Supplement: Supplementary file 3 — Additional file 3. [file 12874_2021_1495_MOESM3_ESM.docx]

# **Additional file 3: Supplementary simulation study results not provided in manuscript**

[**Supplementary Table 6:** Simulation results for 15% missing covariates, generated from an independent missing mechanism, with an observed exposure-outcome association 3](#_Toc91851148)

[**Supplementary Table 7:** Simulation results for 30% missing covariates, generated from an independent missing mechanism, with an observed exposure-outcome association 4](#_Toc91851149)

[**Supplementary Table 8:** Simulation results for 15% missing covariates, generated from an independent missing mechanism, with an enhanced exposure-outcome association 5](#_Toc91851150)

[**Supplementary Table 9:** Simulation results for 30% missing covariates, generated from an independent missing mechanism, with an enhanced exposure-outcome association 6](#_Toc91851151)

[**Supplementary Table 10:** Simulation results for 15% missing covariates, generated from an observed dependent missing mechanism, with an observed exposure-outcome association 7](#_Toc91851152)

[**Supplementary Table 11:** Simulation results for 30% missing covariates, generated from an observed dependent missing mechanism, with an observed exposure-outcome association 8](#_Toc91851153)

[**Supplementary Table 12:** Simulation results for 15% missing covariates, generated from an observed dependent missing mechanism, with an enhanced exposure-outcome association 9](#_Toc91851154)

[**Supplementary Table 13:** Simulation results for 30% missing covariates, generated from an observed dependent missing mechanism, with an enhanced exposure-outcome association 10](#_Toc91851155)

[**Supplementary Table 14:** Simulation results for 15% missing covariates, generated from an enhanced dependent missing mechanism, with an observed exposure-outcome association 11](#_Toc91851156)

[**Supplementary Table 15:** Simulation results for 30% missing covariates, generated from an enhanced dependent missing mechanism, with an observed exposure-outcome association 12](#_Toc91851157)

[**Supplementary Table 16:** Simulation results for 15% missing covariates, generated from an enhanced dependent missing mechanism, with an enhanced exposure-outcome association 13](#_Toc91851158)

[**Supplementary Table 17:** Simulation results for 30% missing covariates, generated from an enhanced dependent missing mechanism, with an enhanced exposure-outcome association 14](#_Toc91851159)

Additional file 3 provides the supplementary results for the remaining 72 scenarios not presented in the main manuscript. Each table provides the performance measures of convergence rate, bias and relative bias, empirical standard error, relative error in the model-based standard error and the coverage probability of the 95% confidence interval, for the complete-data analysis, complete-case analysis and the 7 multiple imputation approaches for 6 scenarios (3x subcohort selection probability and 2x estimand).

- Tables S6-S9 provides these results for the above 6 scenarios under the independent missing data mechanism, with the first table showing results for 15% missing and an observed exposure-outcome association, followed by 30% missing with an observed association, the remaining two tables are 15% and 30% missing, respectively, under the enhanced association.
- Tables S10-S13 provide results under the observed dependent missing data mechanism (in the same order).
- Tables S14-S17 provide results under the enhanced dependent missing data mechanism.

**Supplementary Table 6:** Simulation results for 15% missing covariates, generated from an independent missing mechanism, with an observed exposure-outcome association

| **Subcohort Selection** | **Method** |  | **Modified Poisson Model** | | | | | |  | **Logistic Model** | | | | | |
| --- | --- | --- | --- | --- | --- | --- | --- | --- | --- | --- | --- | --- | --- | --- | --- |
|  |  |  | **Convergence %** | **Bias (relative bias* %)** | | **Empirical SE** | **Relative error in SE %** | **Coverage %** |  | **Convergence %** | **Bias (relative bias* %)** | | **Empirical SE** | **Relative error in SE %** | **Coverage %** |
| Pr = 0.2 | *Complete-data* |  | 100.0 | -0.01 | (-4.19) | 0.31 | -2.24 | 95.30 |  | 100.0 | 0.00 | (-0.28) | 0.35 | -2.12 | 94.95 |
|  | *CCA* |  | 100.0 | -0.01 | (-7.45) | 0.34 | -2.22 | 94.65 |  | 100.0 | 0.00 | (-0.53) | 0.38 | -2.91 | 95.20 |
|  | *FCS-WO* |  | 100.0 | -0.01 | (-4.47) | 0.31 | -1.69 | 95.25 |  | 100.0 | 0.00 | (0.01) | 0.35 | -1.76 | 95.10 |
|  | *FCS-WM* |  | 100.0 | -0.01 | (-4.03) | 0.31 | -1.84 | 95.25 |  | 100.0 | 0.00 | (0.05) | 0.35 | -2.02 | 94.90 |
|  | *FCS-WX* |  | 99.6 | -0.01 | (-5.0) | 0.31 | -1.59 | 95.20 |  | 99.6 | 0.00 | (-0.45) | 0.35 | -1.89 | 94.95 |
|  | *FCS-SS* |  | 99.9 | -0.01 | (-5.55) | 0.31 | -1.59 | 95.30 |  | 99.8 | 0.00 | (-1.28) | 0.35 | -1.90 | 94.90 |
|  | *MVNI-WO* |  | 100.0 | -0.01 | (-4.29) | 0.31 | -1.77 | 95.25 |  | 100.0 | 0.00 | (-0.02) | 0.35 | -1.83 | 94.95 |
|  | *MVNI-WX* |  | 100.0 | -0.01 | (-4.10) | 0.31 | -1.59 | 95.35 |  | 100.0 | 0.00 | (0.03) | 0.35 | -1.95 | 94.95 |
|  | *MVNI-SS* |  | 100.0 | -0.01 | (-4.42) | 0.31 | -1.56 | 95.20 |  | 100.0 | 0.00 | (-0.24) | 0.35 | -1.98 | 94.85 |
| *MC standard errors range* | |  |  | 0.01-0.01 | | 0 - 0.01 | 1.56 - 1.57 | 0.47 - 0.5 |  |  | 0.01-0.01 | | 0.01 - 0.01 | 1.56 - 1.57 | 0.47 - 0.5 |
|  |  |  |  |  |  |  |  |  |  |  |  |  |  |  |  |
| Pr = 0.3 | *Complete-data* |  | 100.0 | 0.00 | (0.80) | 0.28 | -0.70 | 94.75 |  | 100.0 | 0.00 | (2.15) | 0.31 | -0.08 | 95.40 |
|  | *CCA* |  | 100.0 | 0.00 | (3.08) | 0.31 | -0.67 | 95.50 |  | 100.0 | 0.00 | (0.08) | 0.35 | -0.52 | 94.90 |
|  | *FCS-WO* |  | 100.0 | 0.00 | (1.07) | 0.28 | -0.67 | 95.10 |  | 100.0 | 0.00 | (2.38) | 0.32 | -0.25 | 95.60 |
|  | *FCS-WM* |  | 100.0 | 0.00 | (1.01) | 0.28 | -0.70 | 95.15 |  | 100.0 | 0.00 | (2.63) | 0.32 | -0.46 | 95.20 |
|  | *FCS-WX* |  | 99.9 | 0.00 | (0.43) | 0.28 | -0.76 | 95.05 |  | 99.5 | 0.00 | (1.94) | 0.32 | -0.40 | 95.15 |
|  | *FCS-SS* |  | 99.9 | 0.00 | (-0.32) | 0.29 | -0.81 | 95.05 |  | 99.9 | 0.00 | (1.34) | 0.32 | -0.53 | 95.00 |
|  | *MVNI-WO* |  | 100.0 | 0.00 | (1.25) | 0.28 | -0.81 | 95.15 |  | 100.0 | 0.00 | (2.73) | 0.32 | -0.31 | 95.15 |
|  | *MVNI-WX* |  | 100.0 | 0.00 | (1.16) | 0.28 | -0.67 | 95.25 |  | 100.0 | 0.00 | (2.67) | 0.32 | -0.36 | 95.35 |
|  | *MVNI-SS* |  | 100.0 | 0.00 | (1.09) | 0.28 | -0.63 | 95.25 |  | 100.0 | 0.00 | (2.48) | 0.32 | -0.38 | 95.25 |
| *MC standard errors range* | |  |  | 0.01 - 0.01 | | 0 - 0 | 1.58 - 1.58 | 0.46 - 0.50 |  |  | 0.01 - 0.01 | | 0 - 0.01 | 1.58 - 1.59 | 0.46 - 0.49 |
|  |  |  |  |  |  |  |  |  |  |  |  |  |  |  |  |
| Pr = 0.4 | *Complete-data* |  | 100.0 | 0.01 | (5.64) | 0.27 | -0.53 | 94.90 |  | 100.0 | 0.02 | (9.28) | 0.30 | -1.57 | 95.20 |
|  | *CCA* |  | 100.0 | 0.01 | (7.94) | 0.30 | -1.04 | 95.10 |  | 100.0 | 0.02 | (11.48) | 0.33 | -0.79 | 95.30 |
|  | *FCS-WO* |  | 100.0 | 0.01 | (5.73) | 0.27 | -0.81 | 94.45 |  | 100.0 | 0.02 | (9.72) | 0.31 | -1.48 | 95.45 |
|  | *FCS-WM* |  | 100.0 | 0.01 | (5.82) | 0.27 | -0.91 | 94.30 |  | 100.0 | 0.02 | (9.94) | 0.31 | -1.50 | 95.45 |
|  | *FCS-WX* |  | 99.6 | 0.01 | (5.10) | 0.27 | -0.87 | 94.50 |  | 99.6 | 0.02 | (9.21) | 0.31 | -1.49 | 95.45 |
|  | *FCS-SS* |  | 99.7 | 0.01 | (4.70) | 0.27 | -0.95 | 94.55 |  | 99.9 | 0.01 | (8.51) | 0.31 | -1.51 | 95.20 |
|  | *MVNI-WO* |  | 100.0 | 0.01 | (5.82) | 0.27 | -0.88 | 94.45 |  | 100.0 | 0.02 | (9.99) | 0.31 | -1.56 | 95.50 |
|  | *MVNI-WX* |  | 100.0 | 0.01 | (5.63) | 0.27 | -1.04 | 94.30 |  | 100.0 | 0.02 | (9.93) | 0.31 | -1.62 | 95.40 |
|  | *MVNI-SS* |  | 100.0 | 0.01 | (5.75) | 0.27 | -0.91 | 94.40 |  | 100.0 | 0.02 | (9.88) | 0.31 | -1.44 | 95.30 |
| *MC standard errors range* | |  |  | 0.01 - 0.01 | | 0 - 0 | 1.57 - 1.58 | 0.48 - 0.52 |  |  | 0.01 - 0.01 | | 0 - 0.01 | 1.56 - 1.58 | 0.46 - 0.48 |

**Relative bias is the percentage bias relative to the true value used during data generation*

**Supplementary Table 7:** Simulation results for 30% missing covariates, generated from an independent missing mechanism, with an observed exposure-outcome association

| **Subcohort Selection** | **Method** |  | **Modified Poisson Model** | | | | | |  | **Logistic Model** | | | | | |
| --- | --- | --- | --- | --- | --- | --- | --- | --- | --- | --- | --- | --- | --- | --- | --- |
|  |  |  | **Convergence %** | **Bias (relative bias* %)** | | **Empirical SE** | **Relative error in SE %** | **Coverage %** |  | **Convergence %** | **Bias (relative bias* %)** | | **Empirical SE** | **Relative error in SE %** | **Coverage %** |
| Pr = 0.2 | *Complete-data* |  | 100.0 | 0.00 | (-0.66) | 0.31 | -1.73 | 94.85 |  | 100.0 | 0.01 | (7.98) | 0.34 | -0.32 | 95.25 |
|  | *CCA* |  | 100.0 | -0.01 | (-4.04) | 0.38 | -2.18 | 95.30 |  | 100.0 | 0.02 | (14.42) | 0.42 | -1.53 | 94.90 |
|  | *FCS-WO* |  | 100.0 | 0.00 | (-1.54) | 0.31 | -0.80 | 94.70 |  | 100.0 | 0.01 | (8.54) | 0.35 | 0.13 | 95.50 |
|  | *FCS-WM* |  | 100.0 | 0.00 | (-0.80) | 0.31 | -1.21 | 94.60 |  | 100.0 | 0.02 | (9.27) | 0.35 | -0.55 | 95.20 |
|  | *FCS-WX* |  | 98.1 | 0.00 | (-2.43) | 0.31 | -0.86 | 94.65 |  | 98.1 | 0.01 | (7.94) | 0.35 | -0.20 | 95.55 |
|  | *FCS-SS* |  | 98.8 | -0.01 | (-3.80) | 0.31 | -0.88 | 94.85 |  | 98.7 | 0.01 | (6.28) | 0.35 | -0.27 | 95.45 |
|  | *MVNI-WO* |  | 100.0 | 0.00 | (-1.10) | 0.31 | -0.89 | 94.60 |  | 100.0 | 0.01 | (8.79) | 0.35 | 0.11 | 95.30 |
|  | *MVNI-WX* |  | 100.0 | 0.00 | (-1.05) | 0.31 | -0.65 | 94.95 |  | 100.0 | 0.02 | (9.89) | 0.35 | -0.10 | 95.40 |
|  | *MVNI-SS* |  | 100.0 | 0.00 | (-1.28) | 0.31 | -0.58 | 94.85 |  | 100.0 | 0.02 | (9.76) | 0.35 | -0.12 | 95.45 |
| *MC standard errors range* | |  |  | 0.01 - 0.01 | | 0 - 0.01 | 1.56 - 1.58 | 0.47 - 0.51 |  |  | 0.01-0.01 | | 0.01 - 0.01 | 1.57 - 1.59 | 0.46 - 0.49 |
|  |  |  |  |  |  |  |  |  |  |  |  |  |  |  |  |
| Pr = 0.3 | *Complete-data* |  | 100.0 | -0.01 | (-4.57) | 0.28 | 1.00 | 95.65 |  | 100.0 | 0.00 | (-1.67) | 0.32 | -1.75 | 94.85 |
|  | *CCA* |  | 100.0 | 0.00 | (-0.02) | 0.34 | 0.53 | 95.50 |  | 100.0 | -0.01 | (-3.16) | 0.40 | -3.59 | 94.30 |
|  | *FCS-WO* |  | 100.0 | -0.01 | (-5.15) | 0.28 | 1.59 | 96.05 |  | 100.0 | 0.00 | (-0.87) | 0.33 | -1.56 | 95.20 |
|  | *FCS-WM* |  | 100.0 | -0.01 | (-4.96) | 0.28 | 1.34 | 96.00 |  | 100.0 | 0.00 | (-0.73) | 0.33 | -1.90 | 95.20 |
|  | *FCS-WX* |  | 99.0 | -0.01 | (-6.14) | 0.28 | 1.54 | 95.75 |  | 98.9 | 0.00 | (-2.29) | 0.33 | -1.53 | 95.50 |
|  | *FCS-SS* |  | 98.8 | -0.01 | (-7.97) | 0.28 | 1.54 | 95.75 |  | 98.8 | -0.01 | (-3.62) | 0.33 | -1.59 | 95.35 |
|  | *MVNI-WO* |  | 100.0 | -0.01 | (-4.57) | 0.28 | 1.39 | 95.95 |  | 100.0 | 0.00 | (-0.37) | 0.33 | -1.90 | 95.30 |
|  | *MVNI-WX* |  | 100.0 | -0.01 | (-4.55) | 0.28 | 1.64 | 95.55 |  | 100.0 | 0.00 | (-0.40) | 0.32 | -1.63 | 95.35 |
|  | *MVNI-SS* |  | 100.0 | -0.01 | (-4.80) | 0.28 | 1.61 | 95.90 |  | 100.0 | 0.00 | (-0.56) | 0.32 | -1.61 | 95.05 |
| *MC standard errors range* | |  |  | 0.01 - 0.01 | | 0 – 0.01 | 1.60 - 1.62 | 0.44 - 0.46 |  |  | 0.01 - 0.01 | | 0.01 - 0.01 | 1.54 - 1.57 | 0.46 - 0.52 |
|  |  |  |  |  |  |  |  |  |  |  |  |  |  |  |  |
| Pr = 0.4 | *Complete-data* |  | 100.0 | 0.00 | (2.06) | 0.28 | -3.77 | 94.25 |  | 100.0 | 0.00 | (-0.95) | 0.30 | -0.44 | 95.05 |
|  | *CCA* |  | 100.0 | 0.01 | (9.14) | 0.34 | -3.18 | 94.25 |  | 100.0 | 0.00 | (-1.80) | 0.37 | -0.40 | 95.40 |
|  | *FCS-WO* |  | 100.0 | 0.00 | (2.42) | 0.28 | -3.71 | 94.50 |  | 100.0 | 0.00 | (-0.87) | 0.31 | -0.44 | 94.90 |
|  | *FCS-WM* |  | 100.0 | 0.00 | (2.12) | 0.28 | -3.69 | 94.60 |  | 100.0 | 0.00 | (-0.87) | 0.31 | -0.55 | 94.95 |
|  | *FCS-WX* |  | 98.9 | 0.00 | (0.81) | 0.28 | -3.56 | 94.25 |  | 99.0 | 0.00 | (-2.39) | 0.31 | -0.56 | 95.15 |
|  | *FCS-SS* |  | 98.8 | 0.00 | (-0.54) | 0.28 | -3.63 | 94.10 |  | 98.7 | -0.01 | (-3.80) | 0.31 | -0.44 | 95.15 |
|  | *MVNI-WO* |  | 100.0 | 0.00 | (2.67) | 0.28 | -3.92 | 94.35 |  | 100.0 | 0.00 | (-0.75) | 0.31 | -0.69 | 94.85 |
|  | *MVNI-WX* |  | 100.0 | 0.00 | (2.82) | 0.28 | -3.80 | 94.25 |  | 100.0 | 0.00 | (-0.51) | 0.31 | -0.70 | 94.85 |
|  | *MVNI-SS* |  | 100.0 | 0.00 | (2.55) | 0.28 | -3.54 | 94.25 |  | 100.0 | 0.00 | (-0.59) | 0.31 | -0.46 | 94.85 |
| *MC standard errors range* | |  |  | 0.01 - 0.01 | | 0 – 0.01 | 1.53 - 1.55 | 0.51 - 0.53 |  |  | 0.01 - 0.01 | | 0 - 0.01 | 1.58 - 1.59 | 0.47 - 0.49 |

**Relative bias is the percentage bias relative to the true value used during data generation*

**Supplementary Table 8:** Simulation results for 15% missing covariates, generated from an independent missing mechanism, with an enhanced exposure-outcome association

| **Subcohort Selection** | **Method** |  | **Modified Poisson Model** | | | | | |  | **Logistic Model** | | | | | |
| --- | --- | --- | --- | --- | --- | --- | --- | --- | --- | --- | --- | --- | --- | --- | --- |
|  |  |  | **Convergence %** | **Bias (relative bias* %)** | | **Empirical SE** | **Relative error in SE %** | **Coverage %** |  | **Convergence %** | **Bias (relative bias* %)** | | **Empirical SE** | **Relative error in SE %** | **Coverage %** |
| Pr = 0.2 | *Complete-data* |  | 100.0 | 0.02 | (3.28) | 0.27 | -1.98 | 94.65 |  | 100.0 | 0.03 | (4.81) | 0.32 | -1.79 | 94.85 |
|  | *CCA* |  | 100.0 | 0.02 | (3.34) | 0.29 | -2.32 | 94.35 |  | 100.0 | 0.04 | (5.38) | 0.35 | -2.52 | 94.65 |
|  | *FCS-WO* |  | 100.0 | 0.02 | (3.27) | 0.27 | -1.94 | 94.75 |  | 100.0 | 0.04 | (5.17) | 0.32 | -0.84 | 95.35 |
|  | *FCS-WM* |  | 100.0 | 0.02 | (3.51) | 0.27 | -2.23 | 94.80 |  | 100.0 | 0.04 | (5.28) | 0.32 | -1.45 | 95.20 |
|  | *FCS-WX* |  | 99.7 | 0.02 | (3.35) | 0.27 | -2.23 | 94.60 |  | 99.7 | 0.04 | (5.13) | 0.32 | -1.24 | 95.20 |
|  | *FCS-SS* |  | 100.0 | 0.02 | (3.35) | 0.27 | -2.23 | 94.65 |  | 99.9 | 0.04 | (5.09) | 0.32 | -1.27 | 95.15 |
|  | *MVNI-WO* |  | 100.0 | 0.02 | (3.30) | 0.27 | -1.95 | 94.90 |  | 100.0 | 0.04 | (5.17) | 0.32 | -0.98 | 95.35 |
|  | *MVNI-WX* |  | 100.0 | 0.02 | (3.37) | 0.27 | -2.05 | 94.70 |  | 100.0 | 0.04 | (5.15) | 0.32 | -1.14 | 95.20 |
|  | *MVNI-SS* |  | 100.0 | 0.02 | (3.16) | 0.27 | -2.19 | 94.60 |  | 100.0 | 0.04 | (5.07) | 0.32 | -1.25 | 95.10 |
| *MC standard errors range* | |  |  | 0.01 - 0.01 | | 0 - 0 | 1.55 - 1.56 | 0.49 - 0.52 |  |  | 0.01-0.01 | | 0.01 - 0.01 | 1.55 - 1.58 | 0.47 - 0.50 |
|  |  |  |  |  |  |  |  |  |  |  |  |  |  |  |  |
| Pr = 0.3 | *Complete-data* |  | 100.0 | 0.01 | (1.52) | 0.25 | -2.89 | 94.85 |  | 100.0 | 0.03 | (3.81) | 0.29 | -0.04 | 94.80 |
|  | *CCA* |  | 100.0 | 0.01 | (1.63) | 0.27 | -1.98 | 95.95 |  | 100.0 | 0.03 | (4.37) | 0.32 | -0.63 | 94.65 |
|  | *FCS-WO* |  | 100.0 | 0.01 | (1.24) | 0.25 | -2.61 | 95.30 |  | 100.0 | 0.03 | (4.10) | 0.29 | -0.35 | 94.95 |
|  | *FCS-WM* |  | 100.0 | 0.01 | (1.41) | 0.25 | -2.68 | 95.15 |  | 100.0 | 0.03 | (4.21) | 0.29 | -0.49 | 95.00 |
|  | *FCS-WX* |  | 99.8 | 0.01 | (1.27) | 0.25 | -2.66 | 95.25 |  | 99.9 | 0.03 | (4.06) | 0.29 | -0.33 | 95.00 |
|  | *FCS-SS* |  | 100.0 | 0.01 | (1.25) | 0.25 | -2.68 | 95.10 |  | 99.9 | 0.03 | (4.00) | 0.29 | -0.43 | 94.95 |
|  | *MVNI-WO* |  | 100.0 | 0.01 | (1.25) | 0.25 | -2.71 | 95.20 |  | 100.0 | 0.03 | (4.17) | 0.29 | -0.47 | 95.05 |
|  | *MVNI-WX* |  | 100.0 | 0.01 | (1.29) | 0.25 | -2.76 | 95.30 |  | 100.0 | 0.03 | (4.16) | 0.29 | -0.55 | 94.90 |
|  | *MVNI-SS* |  | 100.0 | 0.01 | (1.06) | 0.25 | -2.65 | 95.35 |  | 100.0 | 0.03 | (4.01) | 0.29 | -0.42 | 95.00 |
| *MC standard errors range* | |  |  | 0.01 - 0.01 | | 0 – 0 | 1.54 - 1.56 | 0.44 - 0.49 |  |  | 0.01 - 0.01 | | 0 - 0 | 1.58 - 1.59 | 0.49 - 0.50 |
|  |  |  |  |  |  |  |  |  |  |  |  |  |  |  |  |
| Pr = 0.4 | *Complete-data* |  | 100.0 | 0.02 | (2.41) | 0.23 | -0.09 | 95.30 |  | 100.0 | 0.02 | (2.46) | 0.27 | 1.57 | 95.10 |
|  | *CCA* |  | 100.0 | 0.02 | (2.65) | 0.25 | 0.10 | 95.55 |  | 100.0 | 0.02 | (3.35) | 0.30 | 0.88 | 95.55 |
|  | *FCS-WO* |  | 100.0 | 0.02 | (2.28) | 0.23 | 0.14 | 95.15 |  | 100.0 | 0.02 | (2.65) | 0.27 | 1.71 | 95.05 |
|  | *FCS-WM* |  | 100.0 | 0.02 | (2.36) | 0.23 | 0.06 | 95.25 |  | 100.0 | 0.02 | (2.63) | 0.27 | 1.69 | 95.05 |
|  | *FCS-WX* |  | 99.9 | 0.02 | (2.27) | 0.23 | 0.04 | 95.30 |  | 99.8 | 0.02 | (2.55) | 0.27 | 1.71 | 95.10 |
|  | *FCS-SS* |  | 100.0 | 0.02 | (2.19) | 0.23 | 0.10 | 95.25 |  | 100.0 | 0.02 | (2.49) | 0.27 | 1.68 | 95.10 |
|  | *MVNI-WO* |  | 100.0 | 0.02 | (2.29) | 0.23 | -0.03 | 95.20 |  | 100.0 | 0.02 | (2.63) | 0.27 | 1.61 | 95.00 |
|  | *MVNI-WX* |  | 100.0 | 0.02 | (2.29) | 0.23 | 0.04 | 95.20 |  | 100.0 | 0.02 | (2.61) | 0.27 | 1.75 | 95.25 |
|  | *MVNI-SS* |  | 100.0 | 0.01 | (2.12) | 0.23 | 0.20 | 95.25 |  | 100.0 | 0.02 | (2.56) | 0.27 | 1.68 | 95.10 |
| *MC standard errors range* | |  |  | 0.01 - 0.01 | | 0 – 0 | 1.59 - 1.59 | 0.46 - 0.48 |  |  | 0.01 - 0.01 | | 0 - 0 | 1.60 - 1.61 | 0.46 - 0.49 |

**Relative bias is the percentage bias relative to the true value used during data generation*

**Supplementary Table 9:** Simulation results for 30% missing covariates, generated from an independent missing mechanism, with an enhanced exposure-outcome association

| **Subcohort Selection** | **Method** |  | **Modified Poisson Model** | | | | | |  | **Logistic Model** | | | | | |
| --- | --- | --- | --- | --- | --- | --- | --- | --- | --- | --- | --- | --- | --- | --- | --- |
|  |  |  | **Convergence %** | **Bias (relative bias* %)** | | **Empirical SE** | **Relative error in SE %** | **Coverage %** |  | **Convergence %** | **Bias (relative bias* %)** | | **Empirical SE** | **Relative error in SE %** | **Coverage %** |
| Pr = 0.2 | *Complete-data* |  | 100.0 | 0.02 | (3.49) | 0.27 | -4.46 | 94.15 |  | 100.0 | 0.03 | (4.50) | 0.31 | 0.49 | 95.85 |
|  | *CCA* |  | 100.0 | 0.03 | (4.21) | 0.33 | -4.84 | 93.70 |  | 100.0 | 0.05 | (6.99) | 0.39 | -0.66 | 95.10 |
|  | *FCS-WO* |  | 100.0 | 0.02 | (3.16) | 0.28 | -3.49 | 94.50 |  | 100.0 | 0.03 | (4.95) | 0.32 | 1.25 | 95.60 |
|  | *FCS-WM* |  | 100.0 | 0.02 | (3.59) | 0.28 | -4.37 | 94.05 |  | 100.0 | 0.04 | (5.14) | 0.32 | 0.22 | 95.70 |
|  | *FCS-WX* |  | 98.9 | 0.02 | (3.29) | 0.28 | -3.91 | 94.45 |  | 99.3 | 0.03 | (4.82) | 0.32 | 0.63 | 95.55 |
|  | *FCS-SS* |  | 99.8 | 0.02 | (3.26) | 0.28 | -3.92 | 94.55 |  | 99.8 | 0.03 | (4.63) | 0.32 | 0.70 | 95.50 |
|  | *MVNI-WO* |  | 100.0 | 0.02 | (3.25) | 0.28 | -3.60 | 94.45 |  | 100.0 | 0.03 | (4.89) | 0.32 | 1.10 | 95.75 |
|  | *MVNI-WX* |  | 100.0 | 0.02 | (3.30) | 0.28 | -3.60 | 94.40 |  | 100.0 | 0.03 | (4.96) | 0.32 | 1.28 | 95.75 |
|  | *MVNI-SS* |  | 100.0 | 0.02 | (2.79) | 0.28 | -3.78 | 94.40 |  | 100.0 | 0.03 | (4.67) | 0.32 | 1.00 | 95.45 |
| *MC standard errors range* | |  |  | 0.01 - 0.01 | | 0 – 0.01 | 1.51 - 1.53 | 0.51 - 0.54 |  |  | 0.01-0.01 | | 0 - 0.01 | 1.58 - 1.61 | 0.45 - 0.48 |
|  |  |  |  |  |  |  |  |  |  |  |  |  |  |  |  |
| Pr = 0.3 | *Complete-data* |  | 100.0 | 0.01 | (1.68) | 0.24 | -0.51 | 94.50 |  | 100.0 | 0.03 | (3.94) | 0.29 | -1.57 | 95.68 |
|  | *CCA* |  | 100.0 | 0.02 | (2.27) | 0.29 | -0.80 | 94.80 |  | 100.0 | 0.04 | (5.25) | 0.36 | -1.96 | 94.83 |
|  | *FCS-WO* |  | 100.0 | 0.01 | (1.29) | 0.25 | -0.20 | 94.35 |  | 100.0 | 0.03 | (4.49) | 0.30 | -0.49 | 96.26 |
|  | *FCS-WM* |  | 100.0 | 0.01 | (1.53) | 0.25 | -0.56 | 94.00 |  | 100.0 | 0.03 | (4.60) | 0.30 | -1.12 | 95.84 |
|  | *FCS-WX* |  | 99.3 | 0.01 | (1.32) | 0.25 | -0.35 | 94.10 |  | 99.3 | 0.03 | (4.33) | 0.30 | -0.76 | 95.78 |
|  | *FCS-SS* |  | 99.8 | 0.01 | (1.16) | 0.25 | -0.31 | 94.25 |  | 99.8 | 0.03 | (4.12) | 0.30 | -0.66 | 95.99 |
|  | *MVNI-WO* |  | 100.0 | 0.01 | (1.34) | 0.25 | -0.32 | 94.20 |  | 100.0 | 0.03 | (4.46) | 0.29 | -0.63 | 95.99 |
|  | *MVNI-WX* |  | 100.0 | 0.01 | (1.35) | 0.25 | -0.55 | 94.05 |  | 100.0 | 0.03 | (4.41) | 0.29 | -0.56 | 95.84 |
|  | *MVNI-SS* |  | 100.0 | 0.01 | (0.81) | 0.25 | -0.25 | 94.30 |  | 100.0 | 0.03 | (4.28) | 0.29 | -0.63 | 95.94 |
| *MC standard errors range* | |  |  | 0.01 - 0.01 | | 0 – 0 | 1.58 - 1.58 | 0.50 - 0.53 |  |  | 0.01 - 0.01 | | 0 - 0.01 | 1.60 - 1.62 | 0.44 - 0.51 |
|  |  |  |  |  |  |  |  |  |  |  |  |  |  |  |  |
| Pr = 0.4 | *Complete-data* |  | 100.0 | 0.02 | (2.17) | 0.23 | -1.63 | 94.85 |  | 100.0 | 0.02 | (2.50) | 0.28 | -1.15 | 94.90 |
|  | *CCA* |  | 100.0 | 0.01 | (2.12) | 0.28 | -0.80 | 95.05 |  | 100.0 | 0.03 | (3.86) | 0.34 | -3.62 | 94.35 |
|  | *FCS-WO* |  | 100.0 | 0.01 | (1.65) | 0.24 | -1.89 | 94.90 |  | 100.0 | 0.02 | (2.96) | 0.28 | -1.27 | 95.20 |
|  | *FCS-WM* |  | 100.0 | 0.01 | (1.82) | 0.24 | -1.99 | 95.00 |  | 100.0 | 0.02 | (2.88) | 0.28 | -1.30 | 95.15 |
|  | *FCS-WX* |  | 99.5 | 0.01 | (1.62) | 0.24 | -1.90 | 94.70 |  | 99.2 | 0.02 | (2.63) | 0.28 | -1.28 | 95.15 |
|  | *FCS-SS* |  | 99.8 | 0.01 | (1.49) | 0.24 | -1.76 | 94.75 |  | 99.9 | 0.02 | (2.40) | 0.28 | -1.30 | 95.20 |
|  | *MVNI-WO* |  | 100.0 | 0.01 | (1.61) | 0.24 | -2.11 | 94.80 |  | 100.0 | 0.02 | (2.90) | 0.28 | -1.43 | 95.05 |
|  | *MVNI-WX* |  | 100.0 | 0.01 | (1.64) | 0.24 | -2.00 | 94.60 |  | 100.0 | 0.02 | (2.82) | 0.28 | -1.47 | 95.30 |
|  | *MVNI-SS* |  | 100.0 | 0.01 | (1.27) | 0.24 | -1.56 | 94.85 |  | 100.0 | 0.02 | (2.61) | 0.28 | -1.23 | 95.15 |
| *MC standard errors range* | |  |  | 0.01 - 0.01 | | 0 – 0 | 1.55 - 1.58 | 0.49 - 0.51 |  |  | 0.01 - 0.01 | | 0 – 0.01 | 1.53 - 1.57 | 0.47 - 0.52 |

**Relative bias is the percentage bias relative to the true value used during data generation*

**Supplementary Table 10:** Simulation results for 15% missing covariates, generated from an observed dependent missing mechanism, with an observed exposure-outcome association

| **Subcohort Selection** | **Method** |  | **Modified Poisson Model** | | | | | |  | **Logistic Model** | | | | | |
| --- | --- | --- | --- | --- | --- | --- | --- | --- | --- | --- | --- | --- | --- | --- | --- |
|  |  |  | **Convergence %** | **Bias (relative bias* %)** | | **Empirical SE** | **Relative error in SE %** | **Coverage %** |  | **Convergence %** | **Bias (relative bias* %)** | | **Empirical SE** | **Relative error in SE %** | **Coverage %** |
| Pr = 0.2 | *Complete-data* |  | 100.0 | 0.00 | (2.81) | 0.31 | -3.39 | 94.00 |  | 100.0 | 0.01 | (8.62) | 0.35 | -2.84 | 95.10 |
|  | *CCA* |  | 100.0 | 0.01 | (7.72) | 0.36 | -4.02 | 94.85 |  | 100.0 | 0.02 | (12.38) | 0.39 | -2.04 | 96.00 |
|  | *FCS-WO* |  | 100.0 | 0.00 | (2.76) | 0.32 | -3.38 | 94.45 |  | 100.0 | 0.02 | (9.11) | 0.35 | -2.19 | 95.20 |
|  | *FCS-WM* |  | 100.0 | 0.00 | (2.81) | 0.32 | -3.55 | 94.40 |  | 100.0 | 0.02 | (9.23) | 0.35 | -2.49 | 95.20 |
|  | *FCS-WX* |  | 98.8 | 0.00 | (2.00) | 0.32 | -3.51 | 94.35 |  | 99.1 | 0.01 | (8.26) | 0.35 | -2.20 | 95.35 |
|  | *FCS-SS* |  | 99.6 | 0.00 | (1.42) | 0.32 | -3.49 | 94.30 |  | 99.5 | 0.01 | (7.43) | 0.35 | -2.21 | 95.50 |
|  | *MVNI-WO* |  | 100.0 | 0.00 | (3.02) | 0.32 | -3.41 | 94.35 |  | 100.0 | 0.02 | (9.22) | 0.35 | -2.25 | 95.00 |
|  | *MVNI-WX* |  | 100.0 | 0.00 | (2.93) | 0.32 | -3.42 | 94.45 |  | 100.0 | 0.02 | (9.30) | 0.35 | -2.20 | 95.40 |
|  | *MVNI-SS* |  | 100.0 | 0.00 | (2.70) | 0.32 | -3.40 | 94.30 |  | 100.0 | 0.01 | (8.72) | 0.35 | -2.21 | 95.35 |
| *MC standard errors range* | |  |  | 0.01 - 0.01 | | 0 – 0.01 | 1.53 - 1.54 | 0.49 - 0.53 |  |  | 0.01-0.01 | | 0.01 - 0.01 | 1.54 - 1.56 | 0.44 - 0.49 |
|  |  |  |  |  |  |  |  |  |  |  |  |  |  |  |  |
| Pr = 0.3 | *Complete-data* |  | 100.0 | -0.01 | (-9.44) | 0.28 | -0.41 | 94.95 |  | 100.0 | -0.01 | (-4.44) | 0.32 | -2.05 | 95.15 |
|  | *CCA* |  | 100.0 | -0.01 | (-8.64) | 0.32 | -0.84 | 95.35 |  | 100.0 | 0.00 | (-0.79) | 0.36 | -1.95 | 94.90 |
|  | *FCS-WO* |  | 100.0 | -0.01 | (-9.96) | 0.28 | -0.59 | 94.85 |  | 100.0 | -0.01 | (-4.48) | 0.32 | -1.56 | 95.00 |
|  | *FCS-WM* |  | 100.0 | -0.01 | (-9.62) | 0.28 | -0.72 | 94.95 |  | 100.0 | -0.01 | (-4.28) | 0.32 | -1.75 | 95.20 |
|  | *FCS-WX* |  | 99.6 | -0.02 | (-10.35) | 0.28 | -0.70 | 94.90 |  | 99.5 | -0.01 | (-5.04) | 0.32 | -1.63 | 94.90 |
|  | *FCS-SS* |  | 99.6 | -0.02 | (-11.00) | 0.28 | -0.47 | 95.10 |  | 99.6 | -0.01 | (-5.61) | 0.32 | -1.50 | 95.10 |
|  | *MVNI-WO* |  | 100.0 | -0.01 | (-9.61) | 0.28 | -0.69 | 95.05 |  | 100.0 | -0.01 | (-4.10) | 0.32 | -1.79 | 95.00 |
|  | *MVNI-WX* |  | 100.0 | -0.01 | (-9.56) | 0.28 | -0.54 | 94.80 |  | 100.0 | -0.01 | (-4.25) | 0.32 | -1.71 | 95.05 |
|  | *MVNI-SS* |  | 100.0 | -0.02 | (-10.16) | 0.28 | -0.22 | 95.20 |  | 100.0 | -0.01 | (-4.49) | 0.32 | -1.55 | 94.90 |
| *MC standard errors range* | |  |  | 0.01 - 0.01 | | 0 – 0.01 | 1.58 - 1.59 | 0.47 - 0.50 |  |  | 0.01 - 0.01 | | 0.01 - 0.01 | 1.56 - 1.56 | 0.48 - 0.49 |
|  |  |  |  |  |  |  |  |  |  |  |  |  |  |  |  |
| Pr = 0.4 | *Complete-data* |  | 100.0 | 0.00 | (2.04) | 0.27 | -1.54 | 94.35 |  | 100.0 | 0.01 | (3.70) | 0.30 | 0.02 | 95.00 |
|  | *CCA* |  | 100.0 | 0.02 | (10.59) | 0.31 | -1.44 | 94.55 |  | 100.0 | 0.01 | (8.30) | 0.34 | -0.22 | 95.25 |
|  | *FCS-WO* |  | 100.0 | 0.00 | (2.59) | 0.27 | -1.44 | 94.10 |  | 100.0 | 0.01 | (3.56) | 0.30 | -0.34 | 94.60 |
|  | *FCS-WM* |  | 100.0 | 0.00 | (2.80) | 0.27 | -1.53 | 94.10 |  | 100.0 | 0.01 | (3.48) | 0.30 | -0.32 | 94.50 |
|  | *FCS-WX* |  | 99.7 | 0.00 | (2.05) | 0.27 | -1.52 | 93.90 |  | 99.6 | 0.01 | (3.24) | 0.30 | -0.32 | 94.55 |
|  | *FCS-SS* |  | 99.6 | 0.00 | (0.96) | 0.27 | -1.42 | 94.10 |  | 99.5 | 0.00 | (2.32) | 0.30 | -0.09 | 94.60 |
|  | *MVNI-WO* |  | 100.0 | 0.00 | (2.86) | 0.27 | -1.47 | 94.10 |  | 100.0 | 0.01 | (3.95) | 0.30 | -0.40 | 94.70 |
|  | *MVNI-WX* |  | 100.0 | 0.00 | (2.84) | 0.27 | -1.59 | 93.90 |  | 100.0 | 0.01 | (3.96) | 0.30 | -0.37 | 94.65 |
|  | *MVNI-SS* |  | 100.0 | 0.00 | (2.27) | 0.27 | -1.31 | 94.00 |  | 100.0 | 0.01 | (3.39) | 0.30 | -0.21 | 94.70 |
| *MC standard errors range* | |  |  | 0.01 - 0.01 | | 0 – 0 | 1.57 - 1.57 | 0.51 - 0.54 |  |  | 0.01 - 0.01 | | 0 – 0.01 | 1.58 - 1.59 | 0.48 - 0.51 |

**Relative bias is the percentage bias relative to the true value used during data generation*

**Supplementary Table 11:** Simulation results for 30% missing covariates, generated from an observed dependent missing mechanism, with an observed exposure-outcome association

| **Subcohort Selection** | **Method** |  | **Modified Poisson Model** | | | | | |  | **Logistic Model** | | | | | |
| --- | --- | --- | --- | --- | --- | --- | --- | --- | --- | --- | --- | --- | --- | --- | --- |
|  |  |  | **Convergence %** | **Bias (relative bias* %)** | | **Empirical SE** | **Relative error in SE %** | **Coverage %** |  | **Convergence %** | **Bias (relative bias* %)** | | **Empirical SE** | **Relative error in SE %** | **Coverage %** |
| Pr = 0.2 | *Complete-data* |  | 100.0 | 0.02 | (12.09) | 0.30 | -0.30 | 94.70 |  | 100.0 | 0.01 | (4.58) | 0.34 | 0.89 | 95.35 |
|  | *CCA* |  | 100.0 | 0.03 | (18.69) | 0.39 | -0.09 | 94.55 |  | 100.0 | 0.02 | (9.19) | 0.44 | -0.45 | 95.55 |
|  | *FCS-WO* |  | 100.0 | 0.02 | (11.32) | 0.31 | 0.15 | 94.85 |  | 100.0 | 0.01 | (3.85) | 0.35 | 0.95 | 95.50 |
|  | *FCS-WM* |  | 100.0 | 0.02 | (11.82) | 0.31 | -0.34 | 94.95 |  | 100.0 | 0.01 | (4.49) | 0.35 | 0.35 | 95.15 |
|  | *FCS-WX* |  | 96.3 | 0.01 | (9.77) | 0.31 | 0.17 | 95.00 |  | 97.7 | 0.00 | (2.59) | 0.35 | 0.88 | 95.40 |
|  | *FCS-SS* |  | 96.7 | 0.01 | (8.40) | 0.31 | 0.04 | 95.10 |  | 97.5 | 0.00 | (1.38) | 0.35 | 0.83 | 95.50 |
|  | *MVNI-WO* |  | 100.0 | 0.02 | (12.22) | 0.31 | -0.22 | 94.65 |  | 100.0 | 0.01 | (4.31) | 0.34 | 0.84 | 95.65 |
|  | *MVNI-WX* |  | 100.0 | 0.02 | (11.73) | 0.31 | 0.34 | 94.95 |  | 100.0 | 0.01 | (3.87) | 0.34 | 1.14 | 95.75 |
|  | *MVNI-SS* |  | 100.0 | 0.02 | (10.38) | 0.30 | 0.73 | 94.90 |  | 100.0 | 0.01 | (3.58) | 0.34 | 1.33 | 95.45 |
| *MC standard errors range* | |  |  | 0.01 - 0.01 | | 0 – 0.01 | 1.59 - 1.60 | 0.48 - 0.51 |  |  | 0.01-0.01 | | 0.01 - 0.01 | 1.59 - 1.61 | 0.45 - 0.48 |
|  |  |  |  |  |  |  |  |  |  |  |  |  |  |  |  |
| Pr = 0.3 | *Complete-data* |  | 100.0 | 0.00 | (-1.96) | 0.28 | -0.88 | 94.90 |  | 100.0 | 0.01 | (4.02) | 0.32 | -1.05 | 94.95 |
|  | *CCA* |  | 100.0 | 0.01 | (3.91) | 0.36 | -0.90 | 95.27 |  | 100.0 | 0.01 | (4.94) | 0.41 | -3.14 | 94.30 |
|  | *FCS-WO* |  | 100.0 | 0.00 | (-1.62) | 0.29 | -0.58 | 95.22 |  | 100.0 | 0.01 | (5.66) | 0.32 | -0.22 | 94.80 |
|  | *FCS-WM* |  | 100.0 | 0.00 | (-1.18) | 0.29 | -0.94 | 95.17 |  | 100.0 | 0.01 | (5.93) | 0.32 | -0.46 | 94.70 |
|  | *FCS-WX* |  | 98.1 | 0.00 | (-2.80) | 0.29 | -0.65 | 95.06 |  | 97.5 | 0.01 | (4.10) | 0.32 | -0.41 | 95.05 |
|  | *FCS-SS* |  | 96.1 | -0.01 | (-4.30) | 0.29 | -0.89 | 95.16 |  | 95.9 | 0.00 | (2.60) | 0.32 | -0.35 | 95.00 |
|  | *MVNI-WO* |  | 100.0 | 0.00 | (-0.50) | 0.29 | -0.85 | 95.27 |  | 100.0 | 0.01 | (6.46) | 0.32 | -0.57 | 94.70 |
|  | *MVNI-WX* |  | 100.0 | 0.00 | (-0.61) | 0.29 | -0.72 | 95.16 |  | 100.0 | 0.01 | (6.21) | 0.32 | -0.33 | 94.80 |
|  | *MVNI-SS* |  | 100.0 | 0.00 | (-1.17) | 0.28 | -0.33 | 95.33 |  | 100.0 | 0.01 | (5.69) | 0.32 | 0.04 | 94.95 |
| *MC standard errors range* | |  |  | 0.01 - 0.01 | | 0 – 0.01 | 1.63 - 1.64 | 0.49 - 0.51 |  |  | 0.01 - 0.01 | | 0.01 - 0.01 | 1.55 - 1.59 | 0.49 - 0.52 |
|  |  |  |  |  |  |  |  |  |  |  |  |  |  |  |  |
| Pr = 0.4 | *Complete-data* |  | 100.0 | 0.00 | (-0.86) | 0.27 | -2.11 | 94.75 |  | 100.0 | 0.01 | (3.26) | 0.30 | -0.32 | 95.50 |
|  | *CCA* |  | 100.0 | 0.00 | (-0.96) | 0.35 | -2.10 | 95.00 |  | 100.0 | 0.01 | (8.15) | 0.39 | -1.20 | 95.30 |
|  | *FCS-WO* |  | 100.0 | 0.00 | (-1.39) | 0.28 | -1.98 | 94.70 |  | 100.0 | 0.01 | (3.82) | 0.31 | 0.42 | 95.50 |
|  | *FCS-WM* |  | 100.0 | 0.00 | (-1.16) | 0.28 | -2.11 | 94.70 |  | 100.0 | 0.01 | (3.74) | 0.31 | 0.38 | 95.55 |
|  | *FCS-WX* |  | 98.0 | 0.00 | (-2.46) | 0.28 | -2.02 | 94.65 |  | 98.8 | 0.00 | (2.32) | 0.30 | 0.74 | 95.65 |
|  | *FCS-SS* |  | 96.0 | -0.01 | (-4.57) | 0.28 | -2.12 | 94.50 |  | 95.9 | 0.00 | (0.57) | 0.31 | 0.53 | 95.60 |
|  | *MVNI-WO* |  | 100.0 | 0.00 | (-1.00) | 0.28 | -2.22 | 94.60 |  | 100.0 | 0.01 | (4.24) | 0.30 | 0.36 | 95.40 |
|  | *MVNI-WX* |  | 100.0 | 0.00 | (-1.03) | 0.28 | -2.14 | 94.65 |  | 100.0 | 0.01 | (4.17) | 0.30 | 0.43 | 95.50 |
|  | *MVNI-SS* |  | 100.0 | 0.00 | (-1.47) | 0.27 | -1.46 | 94.85 |  | 100.0 | 0.01 | (3.56) | 0.30 | 0.68 | 95.70 |
| *MC standard errors range* | |  |  | 0.01 - 0.01 | | 0 – 0.01 | 1.55 - 1.57 | 0.49 - 0.51 |  |  | 0.01 - 0.01 | | 0 – 0.01 | 1.57 - 1.60 | 0.45 - 0.47 |

**Relative bias is the percentage bias relative to the true value used during data generation*

**Supplementary Table 12:** Simulation results for 15% missing covariates, generated from an observed dependent missing mechanism, with an enhanced exposure-outcome association

| **Subcohort Selection** | **Method** |  | **Modified Poisson Model** | | | | | |  | **Logistic Model** | | | | | |
| --- | --- | --- | --- | --- | --- | --- | --- | --- | --- | --- | --- | --- | --- | --- | --- |
|  |  |  | **Convergence %** | **Bias (relative bias* %)** | | **Empirical SE** | **Relative error in SE %** | **Coverage %** |  | **Convergence %** | **Bias (relative bias* %)** | | **Empirical SE** | **Relative error in SE %** | **Coverage %** |
| Pr = 0.2 | *Complete-data* |  | 100.0 | 0.02 | (2.41) | 0.27 | -1.92 | 94.85 |  | 100.0 | 0.04 | (6.16) | 0.32 | -1.96 | 94.45 |
|  | *CCA* |  | 100.0 | 0.03 | (4.58) | 0.30 | -0.87 | 94.90 |  | 100.0 | 0.06 | (8.50) | 0.36 | -1.81 | 94.70 |
|  | *FCS-WO* |  | 100.0 | 0.01 | (2.15) | 0.27 | -1.27 | 95.10 |  | 100.0 | 0.04 | (6.37) | 0.32 | -1.15 | 94.95 |
|  | *FCS-WM* |  | 100.0 | 0.02 | (2.40) | 0.27 | -1.69 | 94.90 |  | 100.0 | 0.04 | (6.43) | 0.32 | -1.55 | 94.80 |
|  | *FCS-WX* |  | 99.8 | 0.02 | (2.22) | 0.27 | -1.38 | 95.05 |  | 99.5 | 0.04 | (6.31) | 0.32 | -1.41 | 94.65 |
|  | *FCS-SS* |  | 99.9 | 0.01 | (2.15) | 0.27 | -1.42 | 95.00 |  | 99.9 | 0.04 | (6.18) | 0.32 | -1.37 | 94.80 |
|  | *MVNI-WO* |  | 100.0 | 0.02 | (2.26) | 0.27 | -1.48 | 94.95 |  | 100.0 | 0.04 | (6.43) | 0.32 | -1.29 | 94.95 |
|  | *MVNI-WX* |  | 100.0 | 0.02 | (2.31) | 0.27 | -1.38 | 95.05 |  | 100.0 | 0.04 | (6.38) | 0.32 | -1.37 | 94.75 |
|  | *MVNI-SS* |  | 100.0 | 0.01 | (1.87) | 0.27 | -1.28 | 94.95 |  | 100.0 | 0.04 | (6.07) | 0.32 | -1.18 | 94.95 |
| *MC standard errors range* | |  |  | 0.01 - 0.01 | | 0 – 0 | 1.56 - 1.58 | 0.48 - 0.49 |  |  | 0.01-0.01 | | 0.01 - 0.01 | 1.56 - 1.57 | 0.49 - 0.51 |
|  |  |  |  |  |  |  |  |  |  |  |  |  |  |  |  |
| Pr = 0.3 | *Complete-data* |  | 100.0 | 0.02 | (2.40) | 0.24 | -0.01 | 95.05 |  | 100.0 | 0.03 | (4.93) | 0.29 | -2.24 | 94.55 |
|  | *CCA* |  | 100.0 | 0.03 | (4.41) | 0.28 | -1.77 | 94.25 |  | 100.0 | 0.05 | (7.15) | 0.33 | -2.45 | 94.45 |
|  | *FCS-WO* |  | 100.0 | 0.02 | (2.49) | 0.24 | -0.09 | 95.05 |  | 100.0 | 0.04 | (5.29) | 0.30 | -1.88 | 94.60 |
|  | *FCS-WM* |  | 100.0 | 0.02 | (2.59) | 0.24 | -0.04 | 95.05 |  | 100.0 | 0.04 | (5.33) | 0.30 | -1.98 | 94.75 |
|  | *FCS-WX* |  | 99.8 | 0.02 | (2.44) | 0.24 | 0.00 | 95.05 |  | 99.7 | 0.04 | (5.20) | 0.30 | -1.78 | 94.75 |
|  | *FCS-SS* |  | 99.9 | 0.02 | (2.38) | 0.24 | -0.13 | 95.00 |  | 99.9 | 0.04 | (5.12) | 0.30 | -1.69 | 94.75 |
|  | *MVNI-WO* |  | 100.0 | 0.02 | (2.55) | 0.24 | -0.17 | 94.95 |  | 100.0 | 0.04 | (5.38) | 0.30 | -1.91 | 94.75 |
|  | *MVNI-WX* |  | 100.0 | 0.02 | (2.49) | 0.24 | -0.04 | 95.00 |  | 100.0 | 0.04 | (5.29) | 0.30 | -1.78 | 94.70 |
|  | *MVNI-SS* |  | 100.0 | 0.02 | (2.17) | 0.24 | -0.03 | 94.90 |  | 100.0 | 0.04 | (5.09) | 0.29 | -1.72 | 94.80 |
| *MC standard errors range* | |  |  | 0.01 - 0.01 | | 0 – 0 | 1.56 - 1.59 | 0.49 - 0.52 |  |  | 0.01 - 0.01 | | 0 - 0.01 | 1.55 - 1.56 | 0.50 - 0.51 |
|  |  |  |  |  |  |  |  |  |  |  |  |  |  |  |  |
| Pr = 0.4 | *Complete-data* |  | 100.0 | 0.01 | (1.48) | 0.24 | -3.58 | 94.75 |  | 100.0 | 0.03 | (3.78) | 0.28 | -1.52 | 95.10 |
|  | *CCA* |  | 100.0 | 0.03 | (3.67) | 0.26 | -2.20 | 94.80 |  | 100.0 | 0.04 | (5.17) | 0.31 | -2.70 | 94.85 |
|  | *FCS-WO* |  | 100.0 | 0.01 | (1.42) | 0.24 | -3.00 | 94.80 |  | 100.0 | 0.03 | (3.89) | 0.28 | -1.45 | 95.25 |
|  | *FCS-WM* |  | 100.0 | 0.01 | (1.46) | 0.24 | -3.02 | 94.60 |  | 100.0 | 0.03 | (3.91) | 0.28 | -1.60 | 95.25 |
|  | *FCS-WX* |  | 99.7 | 0.01 | (1.33) | 0.24 | -2.94 | 94.80 |  | 99.8 | 0.03 | (3.83) | 0.28 | -1.51 | 95.10 |
|  | *FCS-SS* |  | 100.0 | 0.01 | (1.28) | 0.24 | -2.90 | 94.80 |  | 100.0 | 0.03 | (3.70) | 0.28 | -1.36 | 95.20 |
|  | *MVNI-WO* |  | 100.0 | 0.01 | (1.43) | 0.24 | -3.10 | 94.75 |  | 100.0 | 0.03 | (3.99) | 0.28 | -1.61 | 95.10 |
|  | *MVNI-WX* |  | 100.0 | 0.01 | (1.41) | 0.24 | -3.12 | 94.60 |  | 100.0 | 0.03 | (3.99) | 0.28 | -1.55 | 95.00 |
|  | *MVNI-SS* |  | 100.0 | 0.01 | (1.11) | 0.24 | -2.93 | 94.85 |  | 100.0 | 0.03 | (3.71) | 0.28 | -1.35 | 95.35 |
| *MC standard errors range* | |  |  | 0.01 - 0.01 | | 0 – 0 | 1.53 - 1.53 | 0.49 - 0.51 |  |  | 0.01 - 0.01 | | 0 – 0 | 1.54 - 1.57 | 0.47 - 0.49 |

**Relative bias is the percentage bias relative to the true value used during data generation*

**Supplementary Table 13:** Simulation results for 30% missing covariates, generated from an observed dependent missing mechanism, with an enhanced exposure-outcome association

| **Subcohort Selection** | **Method** |  | **Modified Poisson Model** | | | | | |  | **Logistic Model** | | | | | |
| --- | --- | --- | --- | --- | --- | --- | --- | --- | --- | --- | --- | --- | --- | --- | --- |
|  |  |  | **Convergence %** | **Bias (relative bias* %)** | | **Empirical SE** | **Relative error in SE %** | **Coverage %** |  | **Convergence %** | **Bias (relative bias* %)** | | **Empirical SE** | **Relative error in SE %** | **Coverage %** |
| Pr = 0.2 | *Complete-data* |  | 100.0 | 0.01 | (2.13) | 0.27 | -1.77 | 94.30 |  | 100.0 | 0.02 | (2.96) | 0.31 | -0.37 | 95.17 |
|  | *CCA* |  | 100.0 | 0.04 | (6.04) | 0.36 | -5.36 | 93.10 |  | 100.0 | 0.07 | (10.59) | 0.40 | -0.89 | 94.79 |
|  | *FCS-WO* |  | 100.0 | 0.01 | (2.03) | 0.27 | -1.19 | 94.75 |  | 100.0 | 0.03 | (4.06) | 0.32 | 0.26 | 95.01 |
|  | *FCS-WM* |  | 100.0 | 0.02 | (2.42) | 0.28 | -2.16 | 94.75 |  | 100.0 | 0.03 | (4.22) | 0.33 | -0.72 | 94.85 |
|  | *FCS-WX* |  | 97.7 | 0.01 | (2.05) | 0.27 | -1.35 | 94.80 |  | 98.1 | 0.03 | (3.96) | 0.32 | -0.05 | 95.01 |
|  | *FCS-SS* |  | 99.6 | 0.01 | (1.86) | 0.27 | -1.35 | 94.70 |  | 99.5 | 0.03 | (3.64) | 0.32 | -0.12 | 95.01 |
|  | *MVNI-WO* |  | 100.0 | 0.01 | (2.15) | 0.27 | -1.39 | 94.70 |  | 100.0 | 0.03 | (4.22) | 0.32 | 0.05 | 95.17 |
|  | *MVNI-WX* |  | 100.0 | 0.01 | (2.14) | 0.27 | -1.07 | 94.80 |  | 100.0 | 0.03 | (4.13) | 0.32 | 0.01 | 95.17 |
|  | *MVNI-SS* |  | 100.0 | 0.01 | (1.35) | 0.27 | -0.81 | 94.85 |  | 100.0 | 0.02 | (3.51) | 0.32 | 0.06 | 95.11 |
| *MC standard errors range* | |  |  | 0.01 - 0.01 | | 0 – 0.01 | 1.51 - 1.58 | 0.48 - 0.49 |  |  | 0.01-0.01 | | 0.01 - 0.01 | 1.64 - 1.65 | 0.50 - 0.51 |
|  |  |  |  |  |  |  |  |  |  |  |  |  |  |  |  |
| Pr = 0.3 | *Complete-data* |  | 100.0 | 0.02 | (2.46) | 0.24 | 0.46 | 95.10 |  | 100.0 | 0.03 | (3.88) | 0.29 | -0.01 | 95.54 |
|  | *CCA* |  | 100.0 | 0.04 | (5.08) | 0.31 | -0.34 | 94.50 |  | 100.0 | 0.04 | (6.48) | 0.37 | -1.18 | 94.92 |
|  | *FCS-WO* |  | 100.0 | 0.02 | (2.53) | 0.24 | 0.89 | 94.85 |  | 100.0 | 0.03 | (4.48) | 0.29 | 0.20 | 95.38 |
|  | *FCS-WM* |  | 100.0 | 0.02 | (2.78) | 0.24 | 0.74 | 94.55 |  | 100.0 | 0.03 | (4.39) | 0.29 | -0.17 | 95.59 |
|  | *FCS-WX* |  | 98.9 | 0.02 | (2.4) | 0.24 | 0.90 | 94.90 |  | 99.3 | 0.03 | (4.31) | 0.29 | 0.25 | 95.85 |
|  | *FCS-SS* |  | 99.8 | 0.02 | (2.25) | 0.24 | 1.18 | 94.85 |  | 99.5 | 0.03 | (3.96) | 0.29 | 0.42 | 95.54 |
|  | *MVNI-WO* |  | 100.0 | 0.02 | (2.59) | 0.24 | 0.89 | 94.50 |  | 100.0 | 0.03 | (4.52) | 0.29 | 0.08 | 95.33 |
|  | *MVNI-WX* |  | 100.0 | 0.02 | (2.5) | 0.24 | 0.84 | 94.80 |  | 100.0 | 0.03 | (4.49) | 0.29 | 0.18 | 95.74 |
|  | *MVNI-SS* |  | 100.0 | 0.01 | (1.83) | 0.24 | 1.24 | 94.90 |  | 100.0 | 0.03 | (3.93) | 0.29 | 0.57 | 95.74 |
| *MC standard errors range* | |  |  | 0.01 - 0.01 | | 0 – 0 | 1.59 - 1.61 | 0.49 - 0.52 |  |  | 0.01 - 0.01 | | 0 - 0.01 | 1.59 - 1.62 | 0.45 - 0.50 |
|  |  |  |  |  |  |  |  |  |  |  |  |  |  |  |  |
| Pr = 0.4 | *Complete-data* |  | 100.0 | 0.01 | (1.72) | 0.23 | 2.14 | 96.00 |  | 100.0 | 0.02 | (2.79) | 0.27 | -0.32 | 95.60 |
|  | *CCA* |  | 100.0 | 0.04 | (5.15) | 0.29 | 0.63 | 95.20 |  | 100.0 | 0.03 | (4.62) | 0.34 | 0.98 | 96.00 |
|  | *FCS-WO* |  | 100.0 | 0.01 | (1.88) | 0.23 | 2.11 | 95.90 |  | 100.0 | 0.02 | (3.06) | 0.28 | 0.31 | 95.70 |
|  | *FCS-WM* |  | 100.0 | 0.01 | (2.05) | 0.23 | 1.85 | 95.75 |  | 100.0 | 0.02 | (3.14) | 0.28 | 0.13 | 95.55 |
|  | *FCS-WX* |  | 99.1 | 0.01 | (1.73) | 0.23 | 2.30 | 95.95 |  | 99.3 | 0.02 | (2.84) | 0.28 | 0.32 | 95.60 |
|  | *FCS-SS* |  | 99.7 | 0.01 | (1.49) | 0.23 | 2.43 | 95.95 |  | 99.7 | 0.02 | (2.63) | 0.28 | 0.32 | 95.65 |
|  | *MVNI-WO* |  | 100.0 | 0.01 | (1.92) | 0.23 | 2.04 | 95.95 |  | 100.0 | 0.02 | (3.19) | 0.28 | 0.02 | 95.60 |
|  | *MVNI-WX* |  | 100.0 | 0.01 | (1.78) | 0.23 | 2.12 | 95.85 |  | 100.0 | 0.02 | (3.18) | 0.28 | 0.04 | 95.80 |
|  | *MVNI-SS* |  | 100.0 | 0.01 | (1.17) | 0.23 | 2.95 | 95.85 |  | 100.0 | 0.02 | (2.63) | 0.28 | 0.73 | 95.80 |
| *MC standard errors range* | |  |  | 0.01 - 0.01 | | 0 – 0 | 1.60 - 1.63 | 0.49 - 0.51 |  |  | 0.01 - 0.01 | | 0 – 0.01 | 1.58 - 1.61 | 0.44 - 0.46 |

**Relative bias is the percentage bias relative to the true value used during data generation*

**Supplementary Table 14:** Simulation results for 15% missing covariates, generated from an enhanced dependent missing mechanism, with an observed exposure-outcome association

| **Subcohort Selection** | **Method** |  | **Modified Poisson Model** | | | | | |  | **Logistic Model** | | | | | |
| --- | --- | --- | --- | --- | --- | --- | --- | --- | --- | --- | --- | --- | --- | --- | --- |
|  |  |  | **Convergence %** | **Bias (relative bias* %)** | | **Empirical SE** | **Relative error in SE %** | **Coverage %** |  | **Convergence %** | **Bias (relative bias* %)** | | **Empirical SE** | **Relative error in SE %** | **Coverage %** |
| Pr = 0.2 | *Complete-data* |  | 100.0 | 0.01 | (5.52) | 0.32 | -5.11 | 94.10 |  | 100.0 | 0.01 | (8.23) | 0.35 | -4.04 | 93.85 |
|  | *CCA* |  | 100.0 | 0.03 | (16.94) | 0.37 | -6.11 | 93.55 |  | 100.0 | 0.03 | (18.10) | 0.42 | -6.35 | 93.60 |
|  | *FCS-WO* |  | 100.0 | 0.01 | (4.45) | 0.32 | -4.85 | 94.45 |  | 100.0 | 0.01 | (8.70) | 0.36 | -4.38 | 94.05 |
|  | *FCS-WM* |  | 100.0 | 0.01 | (4.94) | 0.32 | -5.15 | 94.45 |  | 100.0 | 0.01 | (8.46) | 0.36 | -4.59 | 93.90 |
|  | *FCS-WX* |  | 98.8 | 0.01 | (4.26) | 0.32 | -4.79 | 94.25 |  | 98.9 | 0.01 | (7.84) | 0.36 | -4.36 | 94.05 |
|  | *FCS-SS* |  | 99.3 | 0.00 | (3.25) | 0.32 | -4.76 | 94.30 |  | 99.0 | 0.01 | (7.33) | 0.36 | -4.33 | 93.60 |
|  | *MVNI-WO* |  | 100.0 | 0.01 | (4.80) | 0.32 | -4.86 | 94.60 |  | 100.0 | 0.02 | (9.15) | 0.36 | -4.51 | 93.95 |
|  | *MVNI-WX* |  | 100.0 | 0.01 | (5.13) | 0.32 | -4.75 | 94.45 |  | 100.0 | 0.01 | (8.79) | 0.36 | -4.35 | 93.85 |
|  | *MVNI-SS* |  | 100.0 | 0.01 | (4.35) | 0.32 | -4.54 | 94.55 |  | 100.0 | 0.01 | (8.31) | 0.36 | -3.96 | 94.05 |
| *MC standard errors range* | |  |  | 0.01 - 0.01 | | 0.01 – 0.01 | 1.50 - 1.52 | 0.51 - 0.55 |  |  | 0.01-0.01 | | 0.01 - 0.01 | 1.49 - 1.53 | 0.53 - 0.55 |
|  |  |  |  |  |  |  |  |  |  |  |  |  |  |  |  |
| Pr = 0.3 | *Complete-data* |  | 100.0 | 0.00 | (0.74) | 0.29 | -2.27 | 95.05 |  | 100.0 | 0.00 | (2.45) | 0.32 | -2.94 | 94.20 |
|  | *CCA* |  | 100.0 | 0.01 | (9.38) | 0.33 | -1.09 | 95.60 |  | 100.0 | 0.02 | (15.09) | 0.37 | -1.94 | 94.71 |
|  | *FCS-WO* |  | 100.0 | 0.00 | (0.85) | 0.29 | -1.86 | 95.00 |  | 100.0 | 0.00 | (2.41) | 0.33 | -2.61 | 94.40 |
|  | *FCS-WM* |  | 100.0 | 0.00 | (0.98) | 0.29 | -2.02 | 94.95 |  | 100.0 | 0.00 | (2.54) | 0.33 | -2.78 | 94.51 |
|  | *FCS-WX* |  | 99.3 | 0.00 | (0.31) | 0.29 | -1.78 | 95.15 |  | 99.6 | 0.00 | (1.73) | 0.33 | -2.61 | 94.56 |
|  | *FCS-SS* |  | 99.4 | 0.00 | (-0.35) | 0.29 | -1.76 | 95.30 |  | 99.2 | 0.00 | (0.33) | 0.33 | -2.69 | 94.61 |
|  | *MVNI-WO* |  | 100.0 | 0.00 | (1.33) | 0.29 | -1.92 | 94.95 |  | 100.0 | 0.00 | (2.91) | 0.33 | -2.80 | 94.61 |
|  | *MVNI-WX* |  | 100.0 | 0.00 | (1.53) | 0.29 | -1.94 | 94.80 |  | 100.0 | 0.00 | (2.93) | 0.33 | -2.77 | 94.56 |
|  | *MVNI-SS* |  | 100.0 | 0.00 | (0.80) | 0.29 | -1.55 | 95.20 |  | 100.0 | 0.00 | (2.02) | 0.33 | -2.47 | 94.76 |
| *MC standard errors range* | |  |  | 0.01 - 0.01 | | 0 – 0.01 | 1.55 - 1.58 | 0.46 - 0.50 |  |  | 0.01 - 0.01 | | 0.01 - 0.01 | 1.56 - 1.58 | 0.50 - 0.53 |
|  |  |  |  |  |  |  |  |  |  |  |  |  |  |  |  |
| Pr = 0.4 | *Complete-data* |  | 100.0 | 0.01 | (3.46) | 0.26 | 1.64 | 95.65 |  | 100.0 | 0.01 | (3.34) | 0.31 | -1.87 | 94.45 |
|  | *CCA* |  | 100.0 | 0.02 | (14.45) | 0.31 | 1.71 | 95.90 |  | 100.0 | 0.02 | (12.11) | 0.35 | -2.57 | 94.30 |
|  | *FCS-WO* |  | 100.0 | 0.01 | (3.55) | 0.27 | 1.63 | 95.65 |  | 100.0 | 0.01 | (3.70) | 0.31 | -2.11 | 94.00 |
|  | *FCS-WM* |  | 100.0 | 0.01 | (3.64) | 0.27 | 1.46 | 95.65 |  | 100.0 | 0.01 | (3.95) | 0.31 | -2.08 | 94.15 |
|  | *FCS-WX* |  | 99.5 | 0.00 | (2.90) | 0.27 | 1.65 | 95.75 |  | 99.3 | 0.01 | (3.34) | 0.31 | -1.89 | 94.20 |
|  | *FCS-SS* |  | 99.2 | 0.00 | (2.26) | 0.27 | 1.72 | 95.70 |  | 99.2 | 0.00 | (2.41) | 0.31 | -2.09 | 93.95 |
|  | *MVNI-WO* |  | 100.0 | 0.01 | (3.81) | 0.27 | 1.39 | 95.75 |  | 100.0 | 0.01 | (4.28) | 0.31 | -2.06 | 94.25 |
|  | *MVNI-WX* |  | 100.0 | 0.01 | (3.73) | 0.27 | 1.46 | 95.60 |  | 100.0 | 0.01 | (4.35) | 0.31 | -2.11 | 94.35 |
|  | *MVNI-SS* |  | 100.0 | 0.01 | (3.59) | 0.26 | 1.90 | 95.55 |  | 100.0 | 0.01 | (3.68) | 0.31 | -1.96 | 94.00 |
| *MC standard errors range* | |  |  | 0.01 - 0.01 | | 0 – 0 | 1.61 - 1.62 | 0.44 - 0.46 |  |  | 0.01 - 0.01 | | 0.01 – 0.01 | 1.55 - 1.56 | 0.51 - 0.53 |

**Relative bias is the percentage bias relative to the true value used during data generation*

**Supplementary Table 15:** Simulation results for 30% missing covariates, generated from an enhanced dependent missing mechanism, with an observed exposure-outcome association

| **Subcohort Selection** | **Method** |  | **Modified Poisson Model** | | | | | |  | **Logistic Model** | | | | | |
| --- | --- | --- | --- | --- | --- | --- | --- | --- | --- | --- | --- | --- | --- | --- | --- |
|  |  |  | **Convergence %** | **Bias (relative bias* %)** | | **Empirical SE** | **Relative error in SE %** | **Coverage %** |  | **Convergence %** | **Bias (relative bias* %)** | | **Empirical SE** | **Relative error in SE %** | **Coverage %** |
| Pr = 0.2 | *Complete-data* |  | 100.0 | -0.01 | (-5.79) | 0.31 | -1.69 | 94.67 |  | 100.0 | 0.01 | (8.20) | 0.34 | -1.27 | 95.10 |
|  | *CCA* |  | 100.0 | 0.01 | (5.38) | 0.41 | -0.84 | 94.77 |  | 100.0 | 0.04 | (24.03) | 0.46 | -2.08 | 95.04 |
|  | *FCS-WO* |  | 100.0 | -0.01 | (-4.72) | 0.31 | -1.68 | 95.14 |  | 100.0 | 0.01 | (9.06) | 0.35 | -0.25 | 95.52 |
|  | *FCS-WM* |  | 100.0 | -0.01 | (-3.94) | 0.31 | -1.85 | 95.14 |  | 100.0 | 0.02 | (9.51) | 0.35 | -0.82 | 95.47 |
|  | *FCS-WX* |  | 95.6 | -0.01 | (-5.56) | 0.31 | -1.33 | 95.35 |  | 95.0 | 0.01 | (7.67) | 0.35 | -0.07 | 95.36 |
|  | *FCS-SS* |  | 94.9 | -0.01 | (-7.03) | 0.31 | -1.36 | 95.04 |  | 94.7 | 0.01 | (6.55) | 0.35 | 0.10 | 95.31 |
|  | *MVNI-WO* |  | 100.0 | -0.01 | (-4.04) | 0.31 | -1.89 | 94.93 |  | 100.0 | 0.02 | (9.88) | 0.35 | -0.55 | 95.31 |
|  | *MVNI-WX* |  | 100.0 | -0.01 | (-4.18) | 0.31 | -1.19 | 95.30 |  | 100.0 | 0.02 | (10.08) | 0.35 | 0.08 | 95.36 |
|  | *MVNI-SS* |  | 100.0 | -0.01 | (-5.29) | 0.31 | -0.31 | 94.88 |  | 100.0 | 0.01 | (8.65) | 0.35 | 0.84 | 95.52 |
| *MC standard errors range* | |  |  | 0.01 - 0.01 | | 0 – 0.01 | 1.60 - 1.63 | 0.48 - 0.52 |  |  | 0.01-0.01 | | 0.01 - 0.01 | 1.62 - 1.66 | 0.48 - 0.50 |
|  |  |  |  |  |  |  |  |  |  |  |  |  |  |  |  |
| Pr = 0.3 | *Complete-data* |  | 100.0 | -0.01 | (-8.54) | 0.28 | -1.28 | 95.00 |  | 100.0 | 0.01 | (5.11) | 0.32 | -2.04 | 94.40 |
|  | *CCA* |  | 100.0 | 0.01 | (8.52) | 0.39 | -3.51 | 94.85 |  | 100.0 | 0.03 | (19.31) | 0.42 | -1.20 | 94.70 |
|  | *FCS-WO* |  | 100.0 | -0.01 | (-8.59) | 0.29 | -0.92 | 95.25 |  | 100.0 | 0.01 | (5.76) | 0.33 | -1.75 | 94.85 |
|  | *FCS-WM* |  | 100.0 | -0.01 | (-8.25) | 0.29 | -1.03 | 95.40 |  | 100.0 | 0.01 | (5.92) | 0.33 | -1.98 | 94.60 |
|  | *FCS-WX* |  | 96.6 | -0.02 | (-10.12) | 0.29 | -0.96 | 95.50 |  | 97.6 | 0.01 | (4.33) | 0.33 | -1.34 | 94.90 |
|  | *FCS-SS* |  | 94.2 | -0.02 | (-11.72) | 0.29 | -0.58 | 95.15 |  | 93.5 | 0.00 | (2.56) | 0.33 | -1.48 | 94.70 |
|  | *MVNI-WO* |  | 100.0 | -0.01 | (-8.08) | 0.29 | -1.11 | 95.25 |  | 100.0 | 0.01 | (6.15) | 0.33 | -1.86 | 94.65 |
|  | *MVNI-WX* |  | 100.0 | -0.01 | (-7.96) | 0.29 | -1.19 | 95.30 |  | 100.0 | 0.01 | (5.75) | 0.32 | -1.13 | 95.00 |
|  | *MVNI-SS* |  | 100.0 | -0.01 | (-8.70) | 0.28 | -0.10 | 95.55 |  | 100.0 | 0.01 | (4.52) | 0.32 | -0.60 | 95.20 |
| *MC standard errors range* | |  |  | 0.01 - 0.01 | | 0 – 0.01 | 1.55 - 1.59 | 0.46 - 0.49 |  |  | 0.01 - 0.01 | | 0.01 - 0.01 | 1.56 - 1.58 | 0.48 - 0.51 |
|  |  |  |  |  |  |  |  |  |  |  |  |  |  |  |  |
| Pr = 0.4 | *Complete-data* |  | 100.0 | -0.01 | (-3.92) | 0.27 | -0.39 | 94.80 |  | 100.0 | 0.00 | (-0.16) | 0.30 | 0.26 | 95.35 |
|  | *CCA* |  | 100.0 | 0.02 | (10.51) | 0.37 | -2.74 | 94.50 |  | 100.0 | 0.02 | (13.68) | 0.40 | -1.27 | 95.30 |
|  | *FCS-WO* |  | 100.0 | -0.01 | (-5.41) | 0.27 | -0.48 | 94.55 |  | 100.0 | 0.00 | (0.84) | 0.30 | 0.40 | 95.25 |
|  | *FCS-WM* |  | 100.0 | -0.01 | (-5.37) | 0.27 | -0.67 | 94.40 |  | 100.0 | 0.00 | (0.24) | 0.30 | 0.44 | 95.30 |
|  | *FCS-WX* |  | 97.8 | -0.01 | (-6.90) | 0.27 | -0.43 | 94.65 |  | 98.7 | 0.00 | (-0.94) | 0.30 | 0.71 | 95.40 |
|  | *FCS-SS* |  | 93.9 | -0.01 | (-8.45) | 0.27 | -0.43 | 94.80 |  | 95.1 | 0.00 | (-2.57) | 0.30 | 0.84 | 95.25 |
|  | *MVNI-WO* |  | 100.0 | -0.01 | (-5.03) | 0.27 | -0.79 | 94.45 |  | 100.0 | 0.00 | (1.26) | 0.30 | 0.35 | 95.25 |
|  | *MVNI-WX* |  | 100.0 | -0.01 | (-4.94) | 0.27 | -0.96 | 94.50 |  | 100.0 | 0.00 | (0.8) | 0.30 | 0.24 | 95.15 |
|  | *MVNI-SS* |  | 100.0 | -0.01 | (-5.67) | 0.27 | 0.18 | 94.75 |  | 100.0 | 0.00 | (0.2) | 0.30 | 1.06 | 95.35 |
| *MC standard errors range* | |  |  | 0.01 - 0.01 | | 0 – 0 | 1.56 - 1.59 | 0.50 - 0.51 |  |  | 0.01 - 0.01 | | 0 – 0.01 | 1.58 - 1.61 | 0.47 - 0.48 |

**Relative bias is the percentage bias relative to the true value used during data generation*

**Supplementary Table 16:** Simulation results for 15% missing covariates, generated from an enhanced dependent missing mechanism, with an enhanced exposure-outcome association

| **Subcohort Selection** | **Method** |  | **Modified Poisson Model** | | | | | |  | **Logistic Model** | | | | | |
| --- | --- | --- | --- | --- | --- | --- | --- | --- | --- | --- | --- | --- | --- | --- | --- |
|  |  |  | **Convergence %** | **Bias (relative bias* %)** | | **Empirical SE** | **Relative error in SE %** | **Coverage %** |  | **Convergence %** | **Bias (relative bias* %)** | | **Empirical SE** | **Relative error in SE %** | **Coverage %** |
| Pr = 0.2 | *Complete-data* |  | 100.0 | 0.02 | (2.99) | 0.26 | -0.26 | 94.95 |  | 100.0 | 0.03 | (5.00) | 0.32 | -1.29 | 95.30 |
|  | *CCA* |  | 100.0 | 0.05 | (7.65) | 0.31 | -1.91 | 94.30 |  | 100.0 | 0.07 | (9.51) | 0.36 | -1.26 | 94.85 |
|  | *FCS-WO* |  | 100.0 | 0.02 | (3.13) | 0.26 | 0.06 | 95.20 |  | 100.0 | 0.04 | (5.07) | 0.32 | -1.54 | 95.05 |
|  | *FCS-WM* |  | 100.0 | 0.02 | (3.33) | 0.27 | -0.28 | 95.10 |  | 100.0 | 0.04 | (5.26) | 0.32 | -1.86 | 95.30 |
|  | *FCS-WX* |  | 99.1 | 0.02 | (3.12) | 0.26 | 0.15 | 95.10 |  | 99.5 | 0.03 | (5.04) | 0.32 | -1.52 | 95.15 |
|  | *FCS-SS* |  | 99.9 | 0.02 | (3.00) | 0.26 | 0.31 | 95.15 |  | 99.9 | 0.03 | (4.98) | 0.32 | -1.41 | 95.30 |
|  | *MVNI-WO* |  | 100.0 | 0.02 | (3.28) | 0.26 | -0.03 | 94.95 |  | 100.0 | 0.04 | (5.28) | 0.32 | -1.58 | 95.20 |
|  | *MVNI-WX* |  | 100.0 | 0.02 | (3.24) | 0.26 | 0.16 | 95.10 |  | 100.0 | 0.04 | (5.19) | 0.32 | -1.45 | 95.10 |
|  | *MVNI-SS* |  | 100.0 | 0.02 | (2.64) | 0.26 | 0.60 | 95.00 |  | 100.0 | 0.03 | (4.79) | 0.32 | -1.24 | 95.20 |
| *MC standard errors range* | |  |  | 0.01 - 0.01 | | 0 – 0 | 1.56 - 1.60 | 0.48 - 0.52 |  |  | 0.01-0.01 | | 0.01 - 0.01 | 1.56 - 1.57 | 0.47 - 0.49 |
|  |  |  |  |  |  |  |  |  |  |  |  |  |  |  |  |
| Pr = 0.3 | *Complete-data* |  | 100.0 | 0.02 | (3.10) | 0.24 | -0.83 | 94.90 |  | 100.0 | 0.02 | (2.44) | 0.29 | -0.35 | 95.20 |
|  | *CCA* |  | 100.0 | 0.05 | (7.37) | 0.28 | -1.04 | 94.85 |  | 100.0 | 0.04 | (5.44) | 0.33 | -1.77 | 95.30 |
|  | *FCS-WO* |  | 100.0 | 0.02 | (3.01) | 0.25 | -0.83 | 94.65 |  | 100.0 | 0.02 | (2.65) | 0.29 | -0.11 | 95.70 |
|  | *FCS-WM* |  | 100.0 | 0.02 | (3.11) | 0.25 | -0.98 | 94.60 |  | 100.0 | 0.02 | (2.67) | 0.29 | -0.22 | 95.70 |
|  | *FCS-WX* |  | 99.2 | 0.02 | (2.93) | 0.25 | -0.85 | 94.85 |  | 99.5 | 0.02 | (2.66) | 0.29 | 0.06 | 95.55 |
|  | *FCS-SS* |  | 99.9 | 0.02 | (2.80) | 0.25 | -0.84 | 94.70 |  | 99.7 | 0.02 | (2.53) | 0.29 | 0.07 | 95.55 |
|  | *MVNI-WO* |  | 100.0 | 0.02 | (3.09) | 0.25 | -0.91 | 94.75 |  | 100.0 | 0.02 | (2.84) | 0.29 | -0.16 | 95.75 |
|  | *MVNI-WX* |  | 100.0 | 0.02 | (2.97) | 0.25 | -0.96 | 94.70 |  | 100.0 | 0.02 | (2.86) | 0.29 | -0.15 | 95.65 |
|  | *MVNI-SS* |  | 100.0 | 0.02 | (2.54) | 0.24 | -0.53 | 94.70 |  | 100.0 | 0.02 | (2.44) | 0.29 | 0.21 | 95.60 |
| *MC standard errors range* | |  |  | 0.01 - 0.01 | | 0 – 0 | 1.57 - 1.58 | 0.49 - 0.51 |  |  | 0.01 - 0.01 | | 0 - 0.01 | 1.56 - 1.59 | 0.45 - 0.48 |
|  |  |  |  |  |  |  |  |  |  |  |  |  |  |  |  |
| Pr = 0.4 | *Complete-data* |  | 100.0 | 0.00 | (0.55) | 0.24 | -3.20 | 94.40 |  | 100.0 | 0.02 | (2.21) | 0.28 | -1.88 | 95.05 |
|  | *CCA* |  | 100.0 | 0.03 | (4.76) | 0.27 | -1.15 | 94.85 |  | 100.0 | 0.03 | (4.96) | 0.32 | -0.91 | 94.90 |
|  | *FCS-WO* |  | 100.0 | 0.00 | (0.56) | 0.24 | -3.54 | 94.30 |  | 100.0 | 0.02 | (2.41) | 0.28 | -1.40 | 94.95 |
|  | *FCS-WM* |  | 100.0 | 0.00 | (0.67) | 0.24 | -3.61 | 94.20 |  | 100.0 | 0.02 | (2.40) | 0.28 | -1.53 | 95.10 |
|  | *FCS-WX* |  | 99.6 | 0.00 | (0.46) | 0.24 | -3.36 | 94.25 |  | 99.6 | 0.02 | (2.34) | 0.28 | -1.55 | 94.95 |
|  | *FCS-SS* |  | 100.0 | 0.00 | (0.32) | 0.24 | -3.35 | 94.15 |  | 99.9 | 0.02 | (2.18) | 0.28 | -1.38 | 95.10 |
|  | *MVNI-WO* |  | 100.0 | 0.00 | (0.65) | 0.24 | -3.65 | 94.20 |  | 100.0 | 0.02 | (2.58) | 0.28 | -1.56 | 94.90 |
|  | *MVNI-WX* |  | 100.0 | 0.00 | (0.54) | 0.24 | -3.69 | 94.20 |  | 100.0 | 0.02 | (2.61) | 0.28 | -1.58 | 95.10 |
|  | *MVNI-SS* |  | 100.0 | 0.00 | (0.11) | 0.24 | -3.08 | 94.35 |  | 100.0 | 0.01 | (2.10) | 0.28 | -1.45 | 94.95 |
| *MC standard errors range* | |  |  | 0.01 - 0.01 | | 0 – 0 | 1.53 - 1.57 | 0.49 - 0.52 |  |  | 0.01 - 0.01 | | 0 – 0.01 | 1.56 - 1.57 | 0.48 - 0.49 |

**Relative bias is the percentage bias relative to the true value used during data generation*

**Supplementary Table 17:** Simulation results for 30% missing covariates, generated from an enhanced dependent missing mechanism, with an enhanced exposure-outcome association

| **Subcohort Selection** | **Method** |  | **Modified Poisson Model** | | | | | |  | **Logistic Model** | | | | | |
| --- | --- | --- | --- | --- | --- | --- | --- | --- | --- | --- | --- | --- | --- | --- | --- |
|  |  |  | **Convergence %** | **Bias (relative bias* %)** | | **Empirical SE** | **Relative error in SE %** | **Coverage %** |  | **Convergence %** | **Bias (relative bias* %)** | | **Empirical SE** | **Relative error in SE %** | **Coverage %** |
| Pr = 0.2 | *Complete-data* |  | 100.0 | 0.03 | (3.71) | 0.27 | -3.27 | 94.27 |  | 100.0 | 0.04 | (6.26) | 0.32 | -1.66 | 95.10 |
|  | *CCA* |  | 100.0 | 0.09 | (12.41) | 0.37 | -5.21 | 93.05 |  | 100.0 | 0.08 | (11.9) | 0.42 | -1.59 | 95.20 |
|  | *FCS-WO* |  | 100.0 | 0.03 | (3.79) | 0.28 | -3.04 | 94.87 |  | 100.0 | 0.05 | (6.71) | 0.33 | -0.88 | 95.05 |
|  | *FCS-WM* |  | 100.0 | 0.03 | (4.22) | 0.28 | -3.62 | 94.42 |  | 100.0 | 0.05 | (6.85) | 0.33 | -1.76 | 95.20 |
|  | *FCS-WX* |  | 98.1 | 0.03 | (3.64) | 0.28 | -3.15 | 94.62 |  | 97.9 | 0.04 | (6.47) | 0.33 | -0.99 | 95.15 |
|  | *FCS-SS* |  | 99.3 | 0.02 | (3.41) | 0.28 | -3.36 | 94.72 |  | 99.0 | 0.04 | (6.38) | 0.33 | -1.11 | 95.10 |
|  | *MVNI-WO* |  | 100.0 | 0.03 | (3.89) | 0.28 | -3.26 | 94.77 |  | 100.0 | 0.05 | (6.79) | 0.33 | -1.02 | 95.25 |
|  | *MVNI-WX* |  | 100.0 | 0.03 | (3.70) | 0.28 | -3.10 | 95.13 |  | 100.0 | 0.05 | (6.77) | 0.33 | -1.07 | 95.15 |
|  | *MVNI-SS* |  | 100.0 | 0.02 | (2.59) | 0.27 | -2.34 | 95.23 |  | 100.0 | 0.04 | (5.96) | 0.32 | -0.66 | 95.15 |
| *MC standard errors range* | |  |  | 0.01 - 0.01 | | 0 – 0.01 | 1.52 - 1.56 | 0.48 - 0.57 |  |  | 0.01-0.01 | | 0.01 - 0.01 | 1.56 - 1.58 | 0.48 - 0.49 |
|  |  |  |  |  |  |  |  |  |  |  |  |  |  |  |  |
| Pr = 0.3 | *Complete-data* |  | 100.0 | 0.00 | (0.54) | 0.24 | -0.08 | 95.10 |  | 100.0 | 0.03 | (3.92) | 0.29 | 0.43 | 94.85 |
|  | *CCA* |  | 100.0 | 0.06 | (8.96) | 0.32 | -0.13 | 94.65 |  | 100.0 | 0.07 | (9.48) | 0.38 | -0.88 | 94.40 |
|  | *FCS-WO* |  | 100.0 | 0.01 | (0.88) | 0.25 | 0.46 | 95.25 |  | 100.0 | 0.03 | (4.33) | 0.29 | 1.62 | 95.10 |
|  | *FCS-WM* |  | 100.0 | 0.01 | (1.13) | 0.25 | 0.13 | 94.90 |  | 100.0 | 0.03 | (4.36) | 0.29 | 1.35 | 94.85 |
|  | *FCS-WX* |  | 98.5 | 0.00 | (0.61) | 0.25 | 0.66 | 95.10 |  | 98.2 | 0.03 | (4.07) | 0.29 | 1.53 | 95.00 |
|  | *FCS-SS* |  | 99.2 | 0.00 | (0.37) | 0.25 | 0.50 | 95.00 |  | 99.0 | 0.03 | (3.70) | 0.29 | 1.70 | 95.20 |
|  | *MVNI-WO* |  | 100.0 | 0.01 | (0.95) | 0.25 | 0.14 | 95.00 |  | 100.0 | 0.03 | (4.48) | 0.29 | 1.43 | 95.00 |
|  | *MVNI-WX* |  | 100.0 | 0.00 | (0.69) | 0.25 | 0.31 | 95.15 |  | 100.0 | 0.03 | (4.28) | 0.29 | 1.29 | 94.80 |
|  | *MVNI-SS* |  | 100.0 | 0.00 | (-0.19) | 0.24 | 1.22 | 95.05 |  | 100.0 | 0.02 | (3.46) | 0.29 | 1.99 | 95.10 |
| *MC standard errors range* | |  |  | 0.01 - 0.01 | | 0 – 0.01 | 1.59 - 1.61 | 0.48 - 0.50 |  |  | 0.01 - 0.01 | | 0 - 0.01 | 1.58 - 1.62 | 0.48 - 0.51 |
|  |  |  |  |  |  |  |  |  |  |  |  |  |  |  |  |
| Pr = 0.4 | *Complete-data* |  | 100.0 | 0.00 | (0.56) | 0.23 | 0.30 | 95.05 |  | 100.0 | 0.03 | (3.95) | 0.28 | -1.86 | 95.15 |
|  | *CCA* |  | 100.0 | 0.04 | (6.44) | 0.31 | -0.13 | 95.30 |  | 100.0 | 0.07 | (9.70) | 0.37 | -2.70 | 95.15 |
|  | *FCS-WO* |  | 100.0 | 0.00 | (0.42) | 0.23 | 0.46 | 95.05 |  | 100.0 | 0.03 | (4.27) | 0.28 | -1.98 | 94.95 |
|  | *FCS-WM* |  | 100.0 | 0.00 | (0.51) | 0.23 | 0.39 | 95.15 |  | 100.0 | 0.03 | (4.35) | 0.29 | -2.31 | 94.95 |
|  | *FCS-WX* |  | 99.0 | 0.00 | (0.15) | 0.23 | 0.67 | 95.20 |  | 98.8 | 0.03 | (4.14) | 0.29 | -1.96 | 95.00 |
|  | *FCS-SS* |  | 99.1 | 0.00 | (-0.15) | 0.23 | 0.89 | 95.45 |  | 99.1 | 0.03 | (3.87) | 0.29 | -2.03 | 94.95 |
|  | *MVNI-WO* |  | 100.0 | 0.00 | (0.48) | 0.23 | 0.27 | 95.05 |  | 100.0 | 0.03 | (4.48) | 0.28 | -2.15 | 95.00 |
|  | *MVNI-WX* |  | 100.0 | 0.00 | (0.20) | 0.23 | 0.36 | 95.20 |  | 100.0 | 0.03 | (4.46) | 0.28 | -2.13 | 95.05 |
|  | *MVNI-SS* |  | 100.0 | 0.00 | (-0.57) | 0.23 | 1.70 | 95.30 |  | 100.0 | 0.03 | (3.68) | 0.28 | -1.65 | 95.10 |
| *MC standard errors range* | |  |  | 0.01 - 0.01 | | 0 – 0 | 1.59 - 1.61 | 0.47 - 0.49 |  |  | 0.01 - 0.01 | | 0 – 0.01 | 1.55 - 1.56 | 0.48 - 0.49 |

**Relative bias is the percentage bias relative to the true value used during data generation*
